# Influence of Elevated Atmospheric Carbon Dioxide on Transcriptional Responses of *Bradyrhizobium japonicum* in the Soybean Rhizoplane

**DOI:** 10.1264/jsme2.ME12190

**Published:** 2013-05-11

**Authors:** Masayuki Sugawara, Michael J. Sadowsky

**Affiliations:** 1 Department of Soil, Water, and Climate, BioTechnology Institute, University of Minnesota, St. Paul, Minnesota 55108 USA; 2 Microbial and Plant Genomics Institute, University of Minnesota, St. Paul, Minnesota 55108 USA

**Keywords:** *Bradyrhizobium*, elevated atmospheric CO_2_, microarray, rhizoplane, soybean

## Abstract

Elevated atmospheric CO_2_ can influence the structure and function of rhizoplane and rhizosphere microorganisms by altering root growth and the quality and quantity of compounds released into the rhizoplane and rhizosphere via root exudation. In these studies we investigated the transcriptional responses of *Bradyrhizobium japonicum* cells growing in the rhizoplane of soybean plants exposed to elevated atmospheric CO_2_. The results of microarray analyses indicated that elevated atmospheric CO_2_ concentration indirectly influenced the expression of a large number of genes in *Bradyrhizobium* attached to soybean roots. In addition, relative to plants and bacteria grown under ambient CO_2_ growth conditions, genes involved in C1 metabolism, denitrification and FixK_2_-associated genes, including those involved in nitrogen fixation, microaerobic respiration, respiratory nitrite reductase, and heme biosynthesis, were significantly up-regulated under conditions of elevated CO_2_ in the rhizosphere. The expression profile of genes involved in lipochitooligosaccharide Nod factor biosynthesis and negative transcriptional regulators of nodulation genes, *nolA* and *nodD*_2_, were also influenced by plant growth under conditions of elevated CO_2_. Taken together, the results of these studies indicate that the growth of soybeans under conditions of elevated atmospheric CO_2_ influences gene expressions in *B. japonicum* in the soybean rhizoplane, resulting in changes to carbon/nitrogen metabolism, respiration, and nodulation efficiency.

The concentration of carbon dioxide (CO_2_) in the atmosphere has increased, mainly due to the burning of fossil fuels and changes in land use. An increase in the concentration of atmospheric CO_2_ can significantly alter the function of terrestrial ecosystems (22, 39) and has been shown to directly increase plant growth and productivity due to the enhancement of CO_2_ fixation rates (6, 39). While the concentration of CO_2_ in soil is thought to be ~10–50 times higher than that found in the atmosphere, chiefly due to microbial respiration (27), below-ground microbial processes can be indirectly affected by elevated atmospheric CO_2_ through increased root growth and rhizodeposition rates, and by changes in the quality and quantity of root exudates (41, 46). Numerous studies have shown that elevated atmospheric CO_2_ leads to changes in microbial processes. Although the results of many of these studies have been contradictory, the processes thus far considered include changes in microbial C and N biomass, microbial number, respiration rates, organic matter decomposition, soil enzyme activity, microbial community composition, and bacteria mediating trace gas emission, such as methane and nitrous oxide (17, 21, 41, 46). Elevated concentrations of atmospheric CO_2_ typically stimulate plant carbon uptake through photosynthesis (10), which may bring additional N demands to support plant growth. If N is limiting, however, this may result in decreased plant and soil N concentrations (33, 40). Therefore, the activities of nitrogen fixation, nitrification, and denitrification processes by soil bacteria would be of great importance to modify the N balance in terrestrial ecosystems. In contrast, legume crops might benefit from increased atmospheric CO_2_, because their symbiotic nitrogen fixation capacity allows them to counter usual N limitations to growth (42, 55). Thus, legume plants become more dependent on symbiotic N_2_-fixing bacteria for increased below-ground N availability. The biomass of several legumes species, including *Acacia* species, alfalfa (*Medicago sativa*), and mungbean (*Vigna radiata*), have been shown to increase under conditions of elevated CO_2_, and increased plant growth has sometime been correlated with enhanced nodulation and nitrogenase activity (7, 50). Elevated atmospheric CO_2_ has also been shown to increase nitrogen fixation activity and nodulation of *Bradyrhizobium* in symbiosis with soybean (*Glycine max*) (38) and dry matter production by 50% (2).

The population structure of *Rhizobium* in the rhizosphere has been shown to be altered in response to elevated atmospheric CO_2_ concentration. Schortemeyer *et al.* (48) observed a doubling of the population of *Rhizobium leguminosarum* bv. *trifolii* in the rhizosphere of white clover (*Trifolium repens*) growing under 600 ppm of CO_2_. In addition, Montealegre *et al.* (36) reported a shift in the community composition and an increase in the competitive nodulation of *R. leguminosarum* bv. *trifolii* as a result of plant growth under an increased atmospheric CO_2_ concentration. These authors postulated that the observed differences in population structure specifically found with the rhizobia might be due to changes in the quality and quantity of root exudates, including flavonoid compounds, which are inducers of the expression of rhizobial nodulation genes. Similarly, we found that the population structure of *Bradyrhizobium japonicum* and *B. elkanii* strains in soybean nodules is altered in response to elevated CO_2_, especially during early soybean growing stages (Sugawara *et al.* unpublished). While elevated atmospheric CO_2_ has been shown to alter the structure of below-ground microbial populations, to our knowledge there have been no reports on the effects of this greenhouse gas on the gene expression of rhizobia, living in the plant rhizoplane and rhizosphere.

The oligonucleotide microarrays that we have developed have been used to examine the global transcriptional responses of *B. japonicum* USDA 110 to growth under osmotic and desiccation stress conditions, when cultured in minimal and rich media or in the presence of various inorganic and organic sulfur compounds, under chemoautotrophic conditions, and in the bacteroid state (12, 13, 15, 53). Based on these analyses, several new and well-characterized *Bradyrhizobium* genes have been shown to be specifically involved in tolerances to physiological stresses or to be responsive to growth conditions (12, 13, 15, 53). Thus, it was thought that this microarray platform may be a useful tool to analyze the transcriptional responses of *B. japonicum* cells growing in the soybean rhizoplane under conditions of elevated atmospheric CO_2_.

The aim of this present study was to obtain a comprehensive understanding of the genetic responses of *B. japonicum* cells to growth in the rhizoplane of soybean plants exposed to an elevated concentration of atmospheric CO_2_. Genome-wide transcriptional analyses of root-attached *B. japonicum* cells indicated that the expression of several classes of *B. japonicum* genes, especially those involved in carbon/nitrogen metabolism, microaerobic respiration, and nodulation, were altered in response to plant growth under elevated CO_2_ conditions versus their responses on plants grown under ambient CO_2_ conditions.

## Materials and Methods

### Bacterial strains, media, and growth conditions

The *B. japonicum* strains used in this study, SFJ14–36 (serogroup 38) and SFJ4–24 (serogroup 123), were isolated from root nodules of soybean plants (*Glycine max* cv. 93B15; Pioneer Hi-Bred) grown under conditions of elevated (550 ppm) or ambient CO_2_ conditions, respectively (Sugawara *et al.* unpublished), at the Soy-FACE facility located in Champaign, IL (Soy-FACE web site; http://soyface.illinois.edu/index.htm). The *B. japonicum* USDA 110 was obtained from the USDA-ARS *Rhizobium* Germplasm Resource Collection in Beltsville, MD. *B. japonicum* cells were grown at 30°C, with shaking at 200 rpm, in arabinose-gluconate (AG) medium (45).

### Soybean seedling growth in hydroponic systems

*B. japonicum* strains USDA 110, SFJ14–36 and SFJ4–24 were grown in 10 ml AG medium until the stationary phase, collected by centrifugation at 8,000×*g* for 10 min, and washed twice with sterilized plant growth medium (16). The resultant cells were re-suspended in 10 mL of the same plant growth medium to a final concentration of approximately 10^9^ cells mL^−1^. Seeds of *G. max* cv. Lambert were immersed in 100% ethanol for 5 sec, surface sterilized in 1% sodium hypochlorite for 5 min, and washed 10 times with sterile distilled water. Sterilized seeds were placed on the surface of 1.5% agar in large (245×245 mm) petri dishes (Corning, Corning, NY, USA), and incubated, on a slope, at 27°C in the dark for 3 d. Empty pipette tip boxes (1–200 μL pipette tips, Tip One; USA Scientific, Ocala, FL, USA) were used to grow soybean seedlings for transcriptome analyses. A small hole was made in one side of the cover for insertion of an aeration tube, such that the cover could stay closed. Seedling growth boxes were sterilized by autoclaving, filled with 300 mL plant growth medium and sterilized aeration tubes were connected to an aeration pump that employed a sterile filter. Germinated soybean seedlings (60–70) were each aseptically transferred into holes in the growth boxes, and 3 mL washed bacterial suspension was added through an empty hole to provide approximately 10^7^ cells mL^−1^ growth medium. Four replicates of seedling growth boxes were used for each CO_2_ treatment. Seedling boxes were covered with aluminum foil, transferred to a plant growth chamber, and the aeration pump (Luft Pump; Oceanic Systems, Dallas, TX, USA) was immediately started. Each pump supplied aeration to three growth boxes at a rate of 1.33 L min^−1^. Germinated seedlings were incubated for 3 d at 27°C in the dark. The elevated CO_2_ concentration (550 ppm) in growth chambers was controlled using an APBA-250E indoor CO_2_ monitor (HORIBA, Kyoto, Japan). To investigate a direct influence of atmospheric elevated CO_2_ on the gene expression of *Bradyrhizobium* cells, 3 mL washed bacterial suspension was added to growth boxes without plant seedlings, and the boxes were incubated under the same conditions as described above.

### Sampling of root-attached *Bradyrhizobium* cells

All of the roots from the four replicates of hydroponic boxes were gently rinsed several times in sterile fresh plant growth medium to remove loosely adhering or non-attached bradyrhizobia. Rinsed roots were randomly cut with sterilized scissors and placed in 500 mL bottles containing 180 mL ice-cold extraction buffer (0.1% Na-pyrophosphate, pH 7.0, and 0.1% Tween 20) and 20 mL stop solution (5% H_2_O-saturated phenol, pH4.3, in 95% ethanol). Bottles were agitated at approximately 220 strokes per min for 30 min using an Eberbach model 6000 horizontal shaker (Eberbach, Ann Arbor, MI, USA). Extracts were filtered through three layers of Miracloth (Calbiochem. La Jolla, CA, USA) to remove plant cells and debris, and filtrates were centrifuged at 8,000×*g* for 12 min. To investigate the direct influence of CO_2_ concentration on transcriptional responses, bradyrhizobia were added to growth boxes without soybean seedlings and 180 mL incubated bacterial suspension was immediately added to 20 mL cold stop solution, and centrifuged at 8,000×*g* for 12 min. The resulting cell pellets from all experiments were immediately flash frozen in liquid nitrogen and stored in the freezer at −80°C until RNA extraction.

### RNA isolation and microarray studies

RNA from frozen cell pellets was extracted using a hot phenol method and digested with DNaseI as previously described (12). RNA was purified MEGAclear kits (Ambion, Austin, TX, USA), and bacterial RNA was separated from the sample using Ambion MicrobeEnrich kits. The cDNA synthesis, Cy-dye labeling, micro-array hybridization, and statistical analysis procedures used were as described by Chang *et al.* (12) and Cytryn *et al.* (13). Signal intensities from four microarray slides for each strain of root-attached cells or culture cells grown under elevated and ambient CO_2_ conditions were analyzed using Gene-Pix Pro 6.0 software (Molecular Devices, Sunnyvale, CA, USA). All microarray data have been deposited in Gene Expression Omnibus (GEO) and are accessible through the GEO web site (http://www.ncbi.nlm.nih.gov/geo/) under accession number GSE23296.

### Quantitative real-time RT-PCR (qRT-PCR)

The qRT-PCR reactions were performed as described by Sugawara *et al.* (52). Gene-specific primers for target and control genes for qRT-PCR analyses are shown in Table 1. Expression values for four biological replicates for each treatment were normalized to the expression level of the *parA* gene, which is a housekeeping control gene (bll0631) in the *B. japonicum* genome (13).

### Quantification of total phenolic compounds in root exudates

Root exudates (10 mL) from hydroponic boxes containing soybean seedlings were sampled after 3 d of incubation and evaporated to dryness using a rotary evaporator. Dried exudates were re-suspended in 250 μL of 100% methanol and the total phenolic content of root exudates was determined using the colorimetric method described by Haase *et al.* (19). Calibration standards were prepared using gallic acid (Sigma, St. Louis, MO, USA) and the average concentration of phenolic compounds in samples was calculated after subtracting the mean value obtained from four individual control samples without seedlings (blanks).

## Results

### The influence of elevated atmospheric CO_2_ on the transcriptional responses of *Bradyrhizobium japonicum* in the soybean rhizoplane

**Fig. 1:**
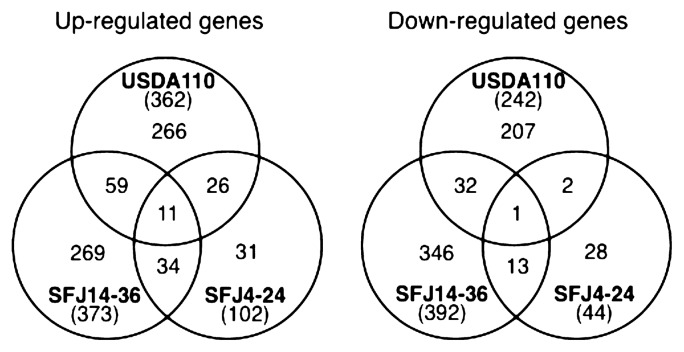
Venn diagram showing numbers of statistically significant up- and down-regulated genes in microarray analyses of mRNA from *Bradyrhizobium japonicum* strains grown in the rhizoplane of soybean plants exposed to atmospheric elevated CO_2_. Values shown are ≥1.5-fold enhanced differential expression in CO_2_-exposed plants relative to those grown under ambient conditions. Numbers shown in parentheses indicate the total number of significantly regulated genes of each strain. Four arrays, representing 8 replicates of each ORF, were analyzed for each strain.

The influence of elevated atmospheric CO_2_ concentration on the transcriptional responses of *B. japonicum* USDA 110 strain in the soybean rhizoplane was investigated by inoculating soybean plants with USDA 110 cells in a hydroponic growth box, and incubating plants under elevated CO_2_ (550 ppm) or ambient CO_2_ (370–380 ppm) conditions. To observe any influences in the initial stages of plant-microbe interactions, young soybean seedlings (3–6 days old) were used. Previous studies have shown that dark CO_2_ fixation is quicker than light-induced CO_2_ fixation in 3–7 days old soybeans (1). Root-attached *Bradyrhizobium* cells were obtained from soybean roots after 3 d of incubation, and total RNA was isolated for microarray analyses. The direct influence of elevated CO_2_ on transcriptional responses in USDA 110 was investigated by incubating *Bradyrhizobium* cells in the same growth box without seedlings under the same CO_2_ concentrations as above. Cells obtained from the box after 3 d of incubation were used for microarray analyses. Signal intensities from four hybridized microarray slides were analyzed and fold-changes of gene expression levels in *Bradyrhizobium* were calculated from cells grown under elevated CO_2_ conditions, relative to those grown under ambient CO_2_ conditions. Results of these studies indicated that when *Bradyrhizobium* was grown in the rhizoplane of plants incubated under conditions of elevated CO_2_, 362 genes in USDA 110 were significantly (*q* value ≤0.05) up- and 242 genes were down-regulated more than 1.5-fold, relative to plants grown under ambient CO_2_ conditions (Fig. 1). In contrast, only 29 genes were differentially regulated (≥1.5-fold) in USDA 110 cells grown in growth boxes without soybean plants under conditions of elevated atmospheric CO_2_, relative to the ambient CO_2_ concentration (Supplementary Table S1). Taken together, the results of these analyses showed that exposure of soybean plants to an elevated concentration of atmospheric CO_2_ led to the induction of genes in *B. japonicum* through soybean roots.

### Transcriptional changes in *B. japonicum* isolate strains in response to elevated CO_2_

The *B. japonicum* strains SFJ14–36 and SFJ4–24 were specifically isolated from root nodules of field-grown soybean plants grown under conditions of elevated (550 ppm) or ambient CO_2_, respectively (Sugawara *et al.* unpublished). In this study, the influence of elevated atmospheric CO_2_ on the transcriptional responses of each strain in the soybean rhizoplane was investigated and compared with both strains and/or standard strain USDA 110 grown under ambient CO_2_ conditions. Results of transcriptional analyses are shown in Fig. 1. A total of 373 and 102 genes were differentially up-regulated more than 1.5-fold, relative to ambient CO_2_ conditions, when strains SFJ14–36 and SFJ4–24, respectively, were grown in the rhizoplane of plants incubated under conditions of elevated CO_2_. In contrast, 392 genes in strains SFJ14–36 and 44 genes in SFJ4–24 were significantly down-regulated (Fig. 1). Thirty-four up- and 13 down-regulated genes were commonly detected in both strains isolated from plants grown under elevated CO_2_. In addition, 59 and 26 up-regulated genes in SFJ14–36 and SFJ4–24, respectively, were also commonly detected in strain USDA 110. Eleven of the genes listed in Table 2 were up-regulated in all the tested strains in response to elevated atmospheric CO_2_. The commonly regulated-genes in the three tested strains included those involved in microaerobic respiration for symbiotic nitrogen fixation (*fixN*, *fixQ*, *fixG*), heme biosynthesis (*hemA*, *hemN*), CO_2_ fixation (*cbbL*), respiratory nitrite reductase (*nirK*), and a two-component response regulator gene (bll2758) located between the *fixLJ* (encoding a low-oxygen responsive two-component regulatory system) and the *fixK*_2_ genes (encoding a transcriptional regulator) involved in symbiotic nitrogen fixation.

**Fig. 2:**
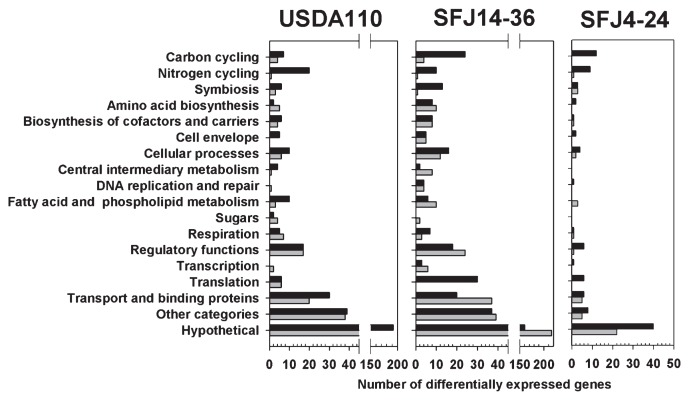
Functional categories of statistically significant, differentially expressed genes in rhizoplane-grown *Bradyrhizobium japonicum* strains. Genes expressed in *B. japonicum* USDA 110, SFJ14–36 and SFJ4–24. Black and gray bars represent up- and down-regulated genes, respectively.

The functional classification of the differentially expressed genes is summarized in Fig. 2. The differentially expressed genes belonging to the carbon cycling, nitrogen cycling, and cellular processes categories were generally more up-regulated in root-attached *B. japonicum* strains grown under elevated CO_2_ conditions than in cells grown under ambient conditions. Included in these categories were genes encoding for CO_2_ fixation (*cbb* genes), a glutathione-dependent formaldehyde oxidation pathway (the *fdhDF*, *flhA* and *gfa genes*), the TCA cycle *nuo* genes, genes involved in denitrification (*nap*, *nir* and *nos*), nitrogen fixation (*fix* genes), and heat-shock chaperonin proteins. Moreover, genes involved in symbiosis with the host plant, including nodulation (*nod*, *nol* and *noe* genes), heme biosynthesis (*hem* genes), and EPS formation (*exo* and *ndv* genes) genes were up-regulated in strain SFJ14–36. In contrast, there was less of an effect of elevated CO_2_ on the expression of genes involved in symbiosis in strains SFJ4–24 and USDA 110 (Fig. 2). Genes annotated as encoding hypothetical proteins comprised about 50% of the differentially expressed genes in each data set. Taken together, the results of these analyses showed that exposure of soybean plants to an elevated concentration of atmospheric CO_2_ led to the induction of genes that are involved in carbon and nitrogen metabolism, and nodulation of soybean in rhizoplane-associating *Bradyrhizobium* strains. These genes are described in more detail below, and all differentially expressed genes are listed in supplementary Table S2.

### Induction of *Bradyrhizobium* genes involved in carbon metabolism in response to elevated CO_2_

Several *B. japonicum* genes involved in carbon cycling were up-regulated in the soybean rhizoplane when plants were grown in the presence of elevated atmospheric CO_2_. Results in Table 3 show that the up-regulated, differentially expressed genes included those involved in CO_2_ fixation (*cbb* genes), the glutathione-dependent formaldehyde oxidation pathway (*fdhDF*, *flhA*, *gfa* genes), and the TCA cycle (*nuo*, *sucA*, *fumC* genes) in strains SFJ14–36 and SFJ4–24. In addition, elevated CO_2_ resulted in the up-regulations of alcohol dehydrogenase genes, and a vanillin:oxygen oxidoreductase gene (*hcaB*) involved in the degradation of methoxy phenolic lignin monomers in both SJ14–36 and USDA 110, and malonate uptake (*mdcLM*), and carbon monoxide dehydrogenase genes in strain SFJ14–36. These results suggested that atmospheric elevated CO_2_ likely led to increased quantities of fixed carbon, including phenolic compounds and alcohol, in root exudates and, in turn, induced the expression of these carbon metabolic genes in *B. japonicum* cells.

### Induction of genes involved in nitrogen cycling in response to elevated CO_2_

*B. japonicum* is thought to be the only *Bradyrhizobium* species that is a true denitrifier (34). When nitrate serves as the terminal electron acceptor and the sole source of nitrogen, this bacterium reduces NO_3_^−^ simultaneously to N_2_ when cultured microaerobically. Genome sequence analysis of *B. japonicum* strain USDA 110 indicated that this bacterium has a complete and functional set of genes for denitrifying NO_3_^−^ to N_2_ (25, 47). The microarray analyses performed here indicated that the denitrification gene *nirK* (encoding the respiratory nitrite reductase), *nap* genes (encoding a periplasmic nitrate reductase), *nos* genes (encoding a nitrous oxide reductase) and *nrt* genes (encoding a nitrate ABC transporter) in strain USDA 110 were significantly up-regulated in response to atmospheric elevated CO_2_ concentration in the soybean rhizoplane. Similarly, up-regulation of *nirK* was also detected in strains SFJ14–36 and SFJ4–24.

Results of quantitative RT-PCR analyses (Table 4), which were used to quantify gene expression of the denitrification genes, indicated that the *nirK* gene was significantly up-regulated in the rhizoplane in response to elevated CO_2_ in all of the tested *B. japonicum* strains. While *norC*, encoding a nitric oxide reductase subunit, was not significantly up-regulated in all tested strains, the expressions of *napE* (encoding a periplasmic nitrate reductase) and *nosZ* (encoding nitrous oxide reductase) significantly changed in strain USDA 110 in response to plant growth under an elevated atmospheric concentration when the strain was grown in the rhizoplane of plants incubated under elevated CO_2_. These results were similar to those found using microarray analyses (Table 3). In contrast, amplification of *nosZ* was not detected in strains SFJ14–36 and SFJ4–24 when using RT-PCR with cDNA as templates (Table 4) or by conventional PCR using genomic DNAs from the same strains (data not shown). This suggests that either strains SFJ14–36 and SFJ4–24 do not possess a *nosZ* gene reacting with the tested primers or they lack this gene in their genomes.

In contrast, the *fixK*_2_ gene, which is a transcriptional regulatory gene involved in symbiotic nitrogen fixation, was induced in *B. japonicum* strains SFJ14–36 and SFJ4–24 in the rhizoplane of soybean plants exposed to elevated CO_2_ (Table 3). Mesa *et al.* (35) identified 51 FixK_2_-associated promoter regions in *B. japonicum* USDA 110 by global transcription and promoter analyses. Interestingly, 53, 23, and 14 of the up-regulated genes in USDA 110, SFJ14–36 and SFJ4–24, respectively, were well correlated with the transcriptionally active FixK_2_-associated genes reported by Mesa *et al.* (35) (Supplementary Table S3). These included genes involved in microaerobic respiration (*fixNOQP* and *fixGHI*), heme biosynthesis (*hemA* and *hemN*), denitrification (*nirK* and *nap* genes), *fixK*_1_ (*fixK*_2_ homolog gene), and *rpoN*_1_ (encoding the RNA polymerase σ^54^ subunit). These results suggested that either enhancement of fixed carbon or other metabolic products could be involved in the activation of FixK_2_ and FixK_2_-regulated genes, and that these genes might play an important role in the adaptation of *B. japonicum* to microaerobic respiration on soybean roots grown under elevated CO_2_.

### Changes in expression of symbiosis-related genes

One of our initial hypotheses was that the expression of genes involved in symbiosis with the host plant might be enhanced by alteration of the quality and quantity of *nod* gene-inducing compounds (*i.e.* flavonoids) exuded in the rhizoplane and rhizosphere of plants grown under elevated atmospheric CO_2_. As shown in Table 3, six nodulation genes (*nodY*, *nodA*, *nodU*, *noeL*, *noeI*, *nolY*) in strain SFJ14–36 were up-regulated >1.5-fold in the soybean rhizoplane of CO_2_-grown plants, relative to that seen in bradyrhizobia recovered from plants grown under ambient CO_2_ conditions. In contrast, *nolA* and *nodD*_2_, which are negative transcriptional regulators of nodulation gene induction, and *nodC*, which is involved in the synthesis of lipochitooligosaccharide Nod factors, were down-regulated in strain SFJ4–24 in response to elevated CO_2_ conditions. The significant down-regulation of the negative transcriptional regulator genes (*nolA* and *nodD*_2_) was verified by qRT-PCR analyses and only occurred in strain SFJ4–24 (Table 4). In contrast, qRT-PCR analyses indicated that both *nodY* and *nodC*, which are both essential for Nod factor biosynthesis in *B. japonicum* (4, 37), were up-regulated in SFJ14–36, but not changed in SFJ4–24 and USDA 110 (Table 4). These results indicated that the induction and repression of nodulation gene expression in response to elevated CO_2_ in the soybean rhizoplane and rhizosphere likely occurs in a strain-specific manner and that more nodulation genes were up-regulated in the strain isolated from soybean nodules grown under elevated CO_2_ conditions than in strains isolated from plants grown under ambient CO_2_ conditions.

Exopolysaccharides (EPS) and cyclic beta-glucans have been shown to provide important functions for N_2_-fixing symbioses (8, 28). Results of the microarray analyses reported here indicated that *ndvC* (encoding a beta glucan synthase), *exoM* (UDP-hexose transferase), blr0562 (encoding a putative polysaccharide deacetylase) in *B. japonicum* strain SFJ14–36, and *exoP* (encoding succinoglycan biosynthesis transporter) in strain SFJ4–24 were up-regulated more than 1.5-fold in response to growth in the rhizoplane of soybean plants exposed to elevated CO_2_, relative to that seen under ambient conditions. These results suggest that *B. japonicum* may be induced to produce polysaccharides in the rhizoplane in response to elevated atmospheric CO_2_, which may be ultimately related to biofilm formation and nodulation functions (18).

### Phenolic content of root exudates

Another of our initial hypotheses was that the quality and quantity of flavonoid (phenolics) root exudates in the rhizoplane and rhizosphere are altered in response to elevated atmospheric CO_2_ (46). This hypothesis appears to be likely correct in the case of common beans (19). Results of analyses performed here indicated that total phenolic compounds, as measured using a colorimetric assay, were significantly greater (*p<*0.05) in root exudates from soybean plants grown under elevated CO_2_ (0.50 μmol g root DW^−1^) than under ambient (0.43 μmol g root DW^−1^) conditions. These results suggest that the production of total phenolic compounds in the soybean rhizoplane and rhizosphere, which include flavonoids and lignins, increase in response to elevated atmospheric CO_2_, and this may lead to the induction of bacterial gene expression in the soybean rhizosphere.

## Discussion

Elevated atmospheric CO_2_ is thought to indirectly affect microbial composition and function by altering root growth and the quality and quantity of fixed carbon released via root exudation (19, 41, 46). Although alteration of the carbon supply due to enhanced CO_2_ fixation rates has been postulated to directly influence nitrogen cycling in soils (21), the influence of this greenhouse gas on the nitrogen-fixing symbiosis between *B. japonicum* and soybean is poorly understood. Recently, however, Prévost *et al.* (38) reported that elevated atmospheric CO_2_ increased soybean nodule number and mass, and increased shoot dry weight, and C and N uptake compared to ambient CO_2_. The reasons, however, for the reported CO_2_-induced enhancement of the symbiosis are currently unknown. It was hypothesized that enhancement of the symbiotic interaction between bradyrhizobia and soybeans was likely due to enhanced plant root growth, water use efficiency, and root exudation, which secondarily influence the growth, colonization, and nodulation of bradyrhizobia growing in the soybean rhizoplane and rhizosphere. In the study reported here, we used transcriptome analyses in order to examine how gene expression in *B. japonicum* cells changed in response to atmospherically elevated CO_2_ in the soybean rhizoplane.

The results of microarray analyses reported here showed that 604 genes in *B. japonicum* USDA 110 were differentially regulated, more than 1.5-fold, relative to ambient CO_2_ conditions, when the bacterium was grown in the rhizoplane of plants incubated under conditions of elevated CO_2_. However, when the USDA 110 cells were grown in plant growth boxes without soybeans, only 29 genes were differentially-regulated (≥1.5-fold) in response to elevated CO_2_. The direct effects of elevated atmospheric CO_2_ concentration on transcription might be included in the 604 differentially regulated genes since the control USDA 110 cells grown in carbon-free plant nutrient solution (without soybeans) are likely starved, with down-regulated transcription of some metabolic genes. However, the number of differentially regulated genes and transcriptional profiles that were observed in bacteria recovered from plants grown under conditions of elevated atmospheric CO_2_ and control conditions were also totally different. Thus, these results suggest that the expression of bradyrhizobial genes is influenced by elevated atmospherically elevated CO_2_ concentration in the soybean rhizoplane and this is likely caused by indirect effects mediated via plant roots.

In strain SFJ14–36, a large number of differentially-regulated genes were detected (765 genes) in response to elevated CO_2_ concentration, relative to those seen in strain SFJ4–24 (146 genes). Results from these analyses suggested that strain SFJ14–36, which was originally isolated from nodules of soybean plants exposed to elevated CO_2_, may be more transcriptionally responsive to the effect of increased carbon dioxide than strain SFJ4–24, which was isolated from soybean nodules of plants exposed to ambient CO_2_; however, the genome structure of either of these *Bradyrhizobium* isolates is likely different from USDA 110, due in large part to the existence of many genomic islands, including the large symbiotic island (25), which are horizontally acquired (14). Itakura *et al.* (23) also showed that some of the genomic islands in strain USDA 110 were missing in the genome of other *B. japonicum* strains. Based on these results, we cannot rule out the possibility that the decrease in the number of differentially expressed genes in strain SFJ4–24 may be due to a different genome structure than strain USDA 110.

### Carbon metabolism

Microarray analyses indicated that carbon metabolism genes in *Bradyrhizobium* that are involved in CO_2_ fixation, the TCA cycle, and a glutathione-dependent formaldehyde oxidation pathway (C1 metabolism) were up-regulated due to plant growth under elevated CO_2_ conditions. While the CO_2_ concentration in soil is ~10–50 times higher than that found in the atmosphere (27), the experiments conducted in this present study were performed under gnotobiotic conditions using a hydroponic system. Thus, CO_2_ was likely limiting for adequate uptake by bradyrhizobia, and induction of CO_2_ fixation genes (*Bradyrhizobium* can grow as an autotroph) may be directly affected by an increased atmospheric (and dissolved) CO_2_ concentration. However, the induction of CO_2_ fixation genes was not detected in USDA 110 cells in response to elevated CO_2_ when the cells incubated in nutrient solution without soybean plants (Supplementary Table S1); therefore, an elevated concentration of atmospheric CO_2_ likely does not directly influence the expression of CO_2_ fixation genes in *Bradyrhizobium*.

The glutathione-dependent formaldehyde oxidation pathway is involved in C1 metabolism and methanol oxidation in *B. japonicum*. Recently, Sudtachat *et al.* (51) reported that *B. japonicum* USDA 110 oxidized methanol, whereas a *mxaF’* mutant did not. Since *mxaF’* (encoding a methanol dehydrogenase) was also up-regulated in strain SFJ14–36, our results suggest that the activity of methanol oxidation to formaldehyde was also likely increased in response to elevated CO_2_. Interestingly *mxaF’* is located upstream of blr6214 (encoding a cytochrome c), *flhA*, and *gfa* in *B. japonicum*, and these four genes form a transcriptional unit (25, 51). Thus, the gene cluster likely functions in methanol oxidation via a glutathione-dependent formaldehyde oxidation pathway. In addition, up-regulation of other alcohol dehydrogenase genes (bll5655, blr3675) in SFJ14–36 was observed following exposure to elevated CO_2_ (Table 3). Rhizobia have previously been shown to grow using ethanol as the sole C source (43), and elevated atmospheric CO_2_ has been shown to increase alcohol production in plants (11). Taken together, these results suggested that the induction of a glutathione-dependent formaldehyde oxidation pathway and alcohol dehydrogenase genes might be caused by an increase in alcohol exudation from soybean roots in response to elevated CO_2_. However, it should be noted that these glutathione-dependent formaldehyde oxidation genes have also been shown to be induced when *B. japonicum* was grown with methoxy phenolic lignin monomers, such as vanillin and vanillate, as sole carbon sources (24, 51). Increased lignin content in wheat was also observed under elevated CO_2_ (9), and Ainsworth *et al.* (3) reported that the expression of soybean lignin biosynthetic genes was up-regulated by elevated CO_2_.

In the study reported here, we observed that the total concentration of phenolic compounds in soybean root exudates, which included lignin monomers, was significantly increased by the growth of plants under elevated CO_2_. This result is consistent with what was previously reported for *Phaseolus vulgaris*, the host plant of *Rhizobium leguminosarum* bv. *phaseoli* (19). Thus, these results suggest that a plausible alternate hypothesis is that the up-regulation of glutathione-dependent formaldehyde oxidation genes might also be due to increased phenolic-lignin content in the root exudates of soybean plants grown under an elevated atmospheric concentration of CO_2_.

### Denitrification and FixK_2_-associated genes

Hartwig *et al.* (20) proposed that increased microbial biomass due to elevated CO_2_ might incorporate more available soil N, result in higher denitrification rates, and thus cause a shortage of available N for plant growth. While some studies have shown that elevated CO_2_ generally results in decreased denitrification rates in soil, contradictory effects have also been reported (5). For example, Barnard *et al.* (5) suggested that CO_2_-induced reduction in denitrification rates was likely a secondary result of a decrease in soil nitrate, which likely results in the reduced availability of electron acceptors for denitrification. *B. japonicum* is thought to be the only *Bradyrhizobium* strain that is a true denitrifier (34), and when nitrate serves as the terminal electron acceptor and the sole source of nitrogen, this bacterium reduces NO_3−_simultaneously to N_2_ when cultured microaerobically. Denitrification in *B. japonicum* USDA 110 depends on the *napEDABC*, *nirK*, *norCBQD* and *nosRZDFYLX* gene clusters, encoding nitrate-, nitrite-, nitric oxide- and nitrous oxide-reductases, respectively (34). The results of transcriptional analyses reported here showed that the expressions of *napE*, *nirK* and *nosZ* genes were induced in strain USDA 110 grown under elevated CO_2_ concentration in the rhizoplane, suggesting that the denitrification rate by *B. japonicum* strain USDA 110 should be enhanced under high CO_2_ concentration; however, this phenomenon might not be true for all bradyrhizobia as the *nos* gene cluster, which encodes N_2_O reductase, was not found in the genomes of other *Bradyrhizobium* strains (23, 47).

The *nirK* gene has also been shown to be involved in respiration and a Cu-containing nitrite reductase in *B. japonicum* has been described (54). Since the transcriptome data showed that *nirK* was significantly induced under elevated CO_2_ conditions in all the tested *Bradyrhizobium* strains, our results suggest that NirK might be enhanced in soybean rhizoplane-localized *B. japonicum* cells in plants exposed to elevated atmospheric CO_2_. The expression of *nirK* is also known to be dependent on FixK_2_, a transcriptional activator of a large group of genes involved in anaerobic and microaerobic metabolism. This allows for bacteroid respiration inside root nodules to support nitrogen fixation activity (35). Results of transcriptome analyses also showed that some FixK_2_-regulated genes, such as those used for microaerobic respiration (*fixNOQP*, *fixGHI*) and heme biosynthesis (*hemN*), were induced in all of the tested strains in response to elevated CO_2_ (Supplementary Table S3). Taken together, these results indicate that enhancement of fixed carbon, or one of its other metabolic products, may be involved in activation of the FixK_2_ regulon, and FixK_2_ might play an important role in the adaptation of *Bradyrhizobium* to conditions conducive to microaerobic respiration that are likely found on soybean roots grown under elevated CO_2_.

### Elevated CO_2_ alters the expression of nodulation genes

The *B. japonicum* nodulation genes (*nod*, *nol*, and *noe*) are positively regulated by NodD_1_, and negatively regulated by NolA and NodD_2_ (32). The *nolA* gene (44), which is induced by chitin and bradyoxetin (29, 31) induces *nodD*_2_ expression, which in turn represses the expression of other nodulation genes (30). The microarray and qRT-PCR analyses reported here indicated that elevated CO_2_ resulted in the enhanced expression of nodulation genes in some, but not all, *Bradyrhizobium* strains growing in the soybean rhizosphere. For example, *nodY* and *nodC*, which are essential for the biosynthesis of Nod factor (4, 37), were significantly up-regulated in strain SFJ14–36, or not changed in strain USDA 110 and SFJ4–24. In contrast, elevated atmospheric CO_2_ resulted in the down-regulation of *nodD*_2_ and *nolA* only in strain SFJ4–24. Since strain SFJ14–36 was isolated from nodules of plants grown under elevated CO_2_ conditions, and *Bradyrhizobium* is reported to have enhanced nodulation on CO_2_-grown plants (38), our data suggest that these growth conditions likely lead to an alteration of the expression of *nod* genes that subsequently results in enhanced nodulation of soybean due to an increase in Nod-factor biosynthesis. This may be due to an indirect effect of a CO_2_-induced enhanced production of *nod* gene inducers (genistein and daidzein) in root exudates in the soybean rhizosphere, which has been shown to occur in both *Glycine max* and *Phaseolus vulgaris* (19, 26). In addition, we show here that total phenolic compounds were increased by growth under elevated CO_2_ conditions.

## Conclusions

Below-ground microbial processes are likely indirectly affected by elevated atmospheric CO_2_ through increased root growth, increases in rhizodeposition rates, enhanced water use efficiency, and changes in the quality and quantity of root exudates released into the rhizosphere. These factors likely strongly influence the physiology and metabolism of microorganisms living in the rhizoplane (and rhizosphere) of plants. The transcriptomic data presented here highlight the physiological and metabolic changes in *B. japonicum* that occur in the soybean rhizoplane in response to plants grown under elevated atmospheric CO_2_ conditions. Overall, our results indicate that elevated atmospheric CO_2_ resulted in changes in the expression of genes involved in carbon/nitrogen metabolism, microaerobic respiration, and nodulation genes in rhizoplane-attached *B. japonicum* cells. While changes in the expression of nodulation genes only occurred in a strain-specific manner, this may be due to variations in the perception and response of individual *B. japonicum* genotypes to plant-released nodulation gene inducers from legume roots. It is our hope that the transcriptome data presented here provide a foundation for future work in studying the genetic and functional responses of micro-organisms in the rhizosphere and their response to anthropogenic changes.

## Supplementary Material



## Figures and Tables

**Table 1:** Quantitative RT-PCR primers used in this study

Gene	Sequence (5′→3′)†		Amplicon size (bp)

	Forward primer	Reverse primer	
*napE*	CAACTGCGTCAAGGGCTACT	GATGTTGTTGGTGCGGAAG	280
*nirK*	GCGACTATGTCTGGGAGACC	TCGTCGTTCCACTTGCCTTC	205
*norC*	TGGGAGAAGAACTCCTGCAT	AGATCGTTGAGCTCCTGGTC	218
*nosZ*	CTGTTCGACGACAAGATCAA	AGCGAGATCAGCCATTTTCC	281
*nodY*	GAGAAGAGCTCCCAGACGTG	GCGTCTGCCTCTATTCTTCG	197
*nodC*	GCTAGACGTTATCGGGCAAA	AGATATTGATCGGCGTGTGG	209
*nodD*_1_	CTGCTGCACATCCAACTCTC	GTTCATCAGAAAATGGCAGC	181
*nodD*_2_	GAGGCTCTGATGCACATTGA	TCTGGCCGGATAACAAAATC	236
*nolA*	ACCTCACTCACGGACTTGCT	GCGAAGATCATCACGGATTC	249
*parA*	GGACGCCGATTACACCTATG	GTAGACCTTCTCGCCCATGA	282

† Nucleotide gene sequences of *B. japonicum* USDA 110 were obtained from GenBank (account number: NC_004463.1) and oligonucleotide primers were designed using Primer3 (http://frodo.wi.mit.edu/).

**Table 2:** Commonly up- and down-regulated genes in *Bradyrhizobium japonicum* strains USDA 110, SFJ14–36, and SFJ4–24 growing in the rhizoplane of soybean plants exposed to elevated atmospheric CO_2_

Locus	Gene	Fold change in expression in strain† (Elevated CO_2_/Ambient CO_2_)			Description

		USDA110	SFJ14–36	SFJ4–24	
bll1200	*hemA*	2.7	2.0	1.6	5-Aminolevulinic acid synthase
blr2585	*cbbL*	1.5	1.6	2.1	Ribulose 1,5-bisphosphate carboxylase/oxygenase large subunit
bll2590	*—*	2.0	2.4	1.6	Hypothetical protein bll2590
bll2758	*—*	1.9	2.0	4.5	Two-component response regulator
blr2763	*fixN*	1.9	1.5	1.8	Cytochrome-c oxidase
bsr2765	*fixQ*	2.9	1.8	2.8	cbb3 oxidase, subunit IV
blr2767	*fixG*	2.3	1.7	4.1	Iron-sulfur cluster-binding protein
bsr6521	*—*	1.8	2.1	2.4	Hypothetical protein bsr6521
blr7089	*nirK*	2.6	1.8	1.7	Respiratory nitrite reductase
bll7086	*hemN*	2.9	2.0	2.6	Anaerobic coproporphyrinogen III oxidase
bll7551	*—*	3.4	2.1	1.9	Hypothetical protein bll7551
blr1067	*—*	−1.8	−9.8	−2.2	ABC transporter ATP-binding protein

† Differentially expressed genes were selected based on a ≥1.5 (or ≤−1.5) -fold induction cutoff with *q* value ≤0.05.

**Table 3:** Significantly regulated carbon and nitrogen cycling and symbiosis-related genes in *Bradyrhizobium japonicum* strains growing in the rhizoplane of soybean plants exposed to elevated atmospheric CO_2_

Locus	Gene	Fold change in strain† (Elevated CO_2_/Ambient CO_2_)			Description

		USDA110	SFJ14–36	SFJ4–24	
**Carbon cycling**					
**CO_2_** **fixation**					
blr2581	*cbbF*	—	2.1	1.7	d-fructose-1,6-bisphosphatase protein
blr2582	*cbbP*	—	2.2	—	Phosphoribulokinase protein
blr2584	*cbbA*	—	1.7	2.2	Fructose-1,6-bisphosphate aldolase protein
blr2585	*cbbL*	1.5	1.6	2.1	Ribulose 1,5-bisphosphate carboxylase/oxygenase large subunit
blr2586	*cbbS*	—	—	2.2	Ribulose 1,5-bisphosphate carboxylase/oxygenase small subunit
blr2587	*cbbX*	—	2.4	2.0	CbbX protein
**C1 and alcohol metabolism**					
blr6213	*mxaF’*	—	2.7	—	Methanol dehydrogenase large subunit-like
blr6215	*flhA*	—	2.2	2.1	Glutathione-dependent formaldehyde dehydrogenase
blr6216	*gfa*	—	3.4	—	Glutathione-dependent formaldehyde-activating enzyme
bll3135	*fdhD*	—	1.6	1.8	Formate dehydrogenase
bll3136	*fdhF*	—	2.0	2.0	Formate dehydrogenase alpha subunit
bll5912	*glyA*	—	2.6	—	Serine hydroxymethyltransferase
bll3998*	*hcaA*	1.8	3.4	—	Vanillin:oxygen oxidoreductase
bll5566		—	2.1	—	Putative sorbitol dehydrogenase
blr3675		−*1.6*	1.6	—	Putative alcohol dehydrogenase
bll4784		—	—	1.5	Aldehyde dehydrogenase
bll5504		—	1.5	—	Putative polyvinyl-alcohol dehydrogenase
bll5655*		1.6	2.6	—	Alcohol dehydrogenase
blr6207	*exaA*	2.0	—	—	Quinoprotein ethanol dehydrogenase
blr0335		—	1.5	—	Putative carbon monoxide dehydrogenase small chain
bll5664	*cooxM*	—	—	2.3	Putative carbon monoxide dehydrogenase medium subunit
**Dicarboxylic acid**					
blr1277	*mdcL*	—	1.6	—	Malonate carrier protein
blr1278	*mdcM*	—	2.0	—	Malonate transporter
**TCA cycle**					
bll0452	*sucA*	1.5	—	—	Alpha-ketoglutarate dehydrogenase
blr2316		—	1.7	—	Probable NADH-ubiquinone oxidoreductase chain F
blr2524		—	—	1.6	Electrotransfer ubiquinone oxidoreductase
bll3137	*nuoF*	—	2.1	—	NADH dehydrogenase I chain F
bll4906	*nuoL*	—	—	1.6	NADH ubiquinone oxidoreductase chain L
bll4909	*nuoI*	−*1.8*	1.6	—	NADH ubiquinone oxidoreductase chain I
bll4917	*nuoC*	—	1.6	—	NADH ubiquinone oxidoreductase chain C
bll6401		—	1.6	—	L-lactate dehydrogenase
blr6519	*fumC*	—	1.6	—	Fumarase C
blr6797		2.3	—	—	Putative citrate lyase
**Nitrogen cycling**					
**Denitrification**					
blr0315	*nosZ*	1.8	—	—	Nitrous oxide reductase
blr0316	*nosD*	1.6	—	—	Periplasmic copper-binding precursor
blr2804	*nrtB*	—	1.5	—	Nitrate ABC transporter permease protein
bll5732	*nrtC*	2.4	—	—	Nitrate ABC transporter ATP-binding protein
bsr7036*	*napE*	2.6	1.7	—	Periplasmic nitrate reductase protein
blr7037*	*napD*	2.7	—	—	Periplasmic nitrate reductase
blr7039*	*napB*	3.2	—	—	Periplasmic nitrate reductase small subunit precursor
blr7040*	*napC*	3.3	—	—	Cytochrome C-type protein
blr7084	*nnrR*	2.1	—	—	FNR/CRP-type transcriptional regulator
blr7089*	*nirK*	2.6	1.8	1.7	Respiratory nitrite reductase
blr7090		2.1	—	—	Probable periplasmic nitrate reductase
Nitrogen fixation					
blr1769	*nifH*	—	1.7	—	Dinitrogenase reductase protein
blr1883*	*rpoN*_1_	1.7	1.6	—	RNA polymerase sigma-54 subunit
bll2757	*fixK*_2_	—	1.6	7.7	Transcriptional regulator, Crp family
blr2763*	*fixN*	1.9	1.5	1.8	Cytochrome-c oxidase
blr2764*	*fixO*	3.5	—	1.6	Cytochrome-c oxidase
bsr2765*	*fixQ*	2.9	1.8	2.8	cbb3 oxidase, subunit IV
blr2766*	*fixP*	4.4	—	2.2	cbb3 oxidase, subunit III
blr2767*	*fixG*	2.3	1.7	4.1	Iron-sulfur cluster-binding protein
blr2768*	*fixH*	3.0	—	2.5	FixH protein
blr2769*	*fixI*	1.7	1.6	—	E1–E2 type cation ATPase
blr5778	*fixG*	2.3	—	—	Nitrogen fixation protein
bll6061*	*fixK*_1_	1.6	—	2.2	Transcriptional regulator, Crp family
**Symbiosis**					
**Nodulation**					
bll1631	*noeL*	—	1.7	—	GDP-mannose 4,6-dehydratase
blr1632	*nodM*	5.9	—	—	Putative glucosamine synthase
bll2016	*nolY*	—	2.0	—	Nodulation protein NolY
blr2024	*nodY*	—	1.6	—	Nodulation protein NodY
blr2025	*nodA*	—	1.9	—	Acyl transferase
blr2029	*nodU*	—	1.8	—	6-O-carbamoyl transferase
blr2034	*nolO*	1.6	—	—	Nodulation protein NolO
blr2062	*noeI*	—	1.6	—	Nodulation protein NoeI
blr1815	*nolV*	−*1.7*	—	—	Nodulation protein NolV
bll2019	*nolA*	1.7	—	−*2.4*	Transcriptional regulator, MerR family
bll2021	*nodD*_2_	—	—	−*2.3*	Transcriptional regulator, LysR family
blr2027	*nodC*	—	—	−*1.5*	Chitin synthase
**Heme synthesis**					
bll1200	*hemA*	2.7	2.0	1.6	5-Aminolevulinic acid synthase
bll2007*	*hemN*_1_	1.8	1.5	—	Coproporphyrinogen III dehydrogenase
bll7086*	*hemN*	2.9	2.0	2.6	Anaerbic coproporphyrinogen III oxidase
**Polysaccharide formation**					
bll2362	*exoP*	—	—	1.7	Succinoglycan biosynthesis transport protein
bll4612	*ndvC*	−*1.5*	1.7	—	Putative beta (1–6) glucans synthase
bll7574	*exoM*	—	1.5	—	UDP-hexose transferase
blr0562		—	1.8	—	Putative polysaccharide deacetylase

† Differentially expressed genes were selected based on a ≥1.5 (or ≤−1.5) -fold induction cutoff with *q* value ≤0.05. —; Not significantly regulated.

* FixK_2_-regulated genes identified by Mesa *et al.* (35).

**Table 4:** Gene expression levels of denitrification and nodulation genes in *Bradyrhizobium* strains grown in the rhizoplane of soybean plants under conditions of elevated atmospheric CO_2_

Gene	Relative expression in strain†		

	USDA110	SFJ14–36	SFJ4–24
**Denitrification**			
*napE*	4.3^*^	1.2	0.9
*nirK*	3.3^*^	2.3^*^	8.3^*^
*norC*	1.2	0.9	1.0
*nosZ*	3.3^*^	ND	ND
**Nodulation**			
*nodY*	1.1	3.9^*^	0.9
*nodC*	1.5	3.5^*^	0.8
*nodD*_1_	0.9	1.3	0.8
*nodD*_2_	1.0	1.2	0.7^*^
*nolA*	1.0	1.2	0.6^*^

† Values determined by quantitative RT-PCR.

The ratio of absolute gene expression value in *B. japonicum* cells in the soybean rhizoplane exposed to elevated atmospheric CO_2_, relative to that of ambient CO_2_ condition. Absolute gene expression values were normalized to the housekeeping gene *parA*. Asterisks indicate a significant difference between elevated CO_2_ condition and ambient condition by ANOVA (*p<*0.05) of four biological replicates. ND: not detected.
