# Supplementary material for: Influence of Elevated Atmospheric Carbon Dioxide on Transcriptional Responses of Bradyrhizobium japonicum in the Soybean Rhizoplane
Source: Microbes Environ. 2013 May 11;28(2):217–27. doi: 10.1264/jsme2.ME12190 (PMC4070659; doi:10.1264/jsme2.ME12190)
Supplement: Supplementary file 1 [file 28_217_s1.pdf]

Table S1. All significantly regulated genes in *Bradyrhizobium japonicum* growing in the rhizoplane of soybean plants exposed to elevated atmospheric CO_2.

	Fold change (Elevated CO_2/Ambient CO_2)			
Locus	USDA 110	SFJ14-36	SFJ4-24	Gene description
**Carbon cycling**				
**CO_2 fixation**				
blr2581		2.1	1.7	putative D-fructose-1,6-bisphosphatase protein (cbbF)
blr2582		2.2		putative phosphoribulokinase protein (cbbP)
blr2584		1.7	2.2	putative fructose-1,6-bisphosphate aldolase protein (cbbA)
blr2585	1.5	1.6	2.1	ribulose 1,5-bisphosphate carboxylase/oxygenase large subunit (cbbL)
blr2586			2.2	ribulose 1,5-bisphosphate carboxylase/oxygenase small subunit (cbbS)
blr2587		2.4	2.0	probable CbbX protein (cbbX)
**C1 and alcohol metabolism**				
blr6213		2.7		methanol dehydrogenase large subunit-like protein (mxaF')
blr6215		2.2	2.1	glutathione-dependent formaldehyde dehydrogenase (flhA)
blr6216		3.4		glutathione-dependent formaldehyde-activating enzyme (gfa)
bll3135		1.6	1.8	formate dehydrogenase (fdhD)
bll3136		2.0	2.0	formate dehydrogenase alpha subunit (fdhF)
bll5566	2.1			putative sorbitol dehydrogenase (EC 1.1.1.14)
bll5912		2.6		serine hydroxymethyltransferase (glyA)
blr3675	-1.6	1.6		putative alcohol dehydrogenase (EC 1.1.1.1)
bll4784			1.5	aldehyde dehydrogenase
bll5504		1.5		putative polyvinyl-alcohol dehydrogenase (EC 1.1.99.23)
bll5655	1.6	2.6		alcohol dehydrogenase
blr6207	2.0			probable quinoprotein ethanol dehydrogenase precursor (EC 1.1.99.-) (exaA)
blr0335		1.5		putative carbon monoxide dehydrogenase small chain
bll5664			2.3	putative carbon monoxide dehydrogenase medium subunit (cooxM)
bll2736	-1.6			putative aldehyde dehydrogenase protein
blr3534		-4.4		putative carbon monoxide dehydrogenase medium chain (EC 1.2.99.2) (cutM)
bll6322	-2.0			aldehyde dehydrogenase
**Dicarboxylic acid**				
blr1277		1.6		malonate carrier protein (mdcL)
blr1278		2.0		malonate transporter (mdcM)
bll0267		-1.6		malonate decarboxylase gamma subunit (mdcE)
bll1273		-1.6		probable malonate decarboxylase (mdcG)
**TCA cycle**				
bll0452	1.5			alpha-ketoglutarate dehydrogenase (sucA)
blr2316		1.7		probable NADH-ubiquinone oxidoreductase chain F (EC 1.6.5.3)
blr2524			1.6	electrotransfer ubiquinone oxidoreductase
bll3137		2.1		NADH dehydrogenase I chain F (nuoF)
bll4906			1.6	NADH ubiquinone oxidoreductase chain L (nuoL)
bll4909	-1.8	1.6		NADH ubiquinone oxidoreductase chain I (nuoI)
bll4917		1.6		NADH ubiquinone oxidoreductase chain C (nuoC)
bll6401		1.6		L-lactate dehydrogenase

blr6519		1.6		fumarase C (fumC)
blr6797	2.3			putative Citrate lyase
bll4919		-3.1		NADH ubiquinone oxidoreductase chain A (nuoA)
**Others**				
bll3998	1.8	3.4		Vanillin:oxygen oxidoreductase (hcaB)
**Nitrogen cycling**				
**Nitrogen fixation**				
blr1769		1.7		dinitrogenase reductase protein (nifH)
bll2757		1.6	7.7	transcriptional regulatory protein Crp family (fixK2)
blr2763	1.9	1.5	1.8	cytochrome-c oxidase (fixN)
blr2764	3.5		1.6	cytochrome-c oxidase (fixO)
bsr2765	2.9	1.8	2.8	cbb3 oxidase, subunit IV (fixQ)
blr2766	4.4		2.2	cbb3 oxidase, subunit III (fixP)
blr2767	2.3	1.7	4.1	iron-sulfur cluster-binding protein (fixG)
blr2768	3.0		2.5	FixH protein (fixH)
blr2769	1.7	1.6		E1-E2 type cation ATPase (fixI)
blr5778	2.3			nitrogen fixation protein (fixG)
bll6061	1.6		2.2	transcriptional regulatory protein Crp family (fixK1)
blr1883	1.7	1.6		RNA polymerase sigma-54 subunit (rpoN1)
bll0800		-3.0	-2.4	Putative nifU protein
bsr1775	-1.7			probable ferredoxin (fixX)
**Denitrification**				
blr0315	1.8			nitrous oxide reductase (nosZ)
blr0316	1.6			periplasmic copper-binding precursor (nosD)
bll5732	2.4			Nitrate ABC transporter ATP-binding protein (nrtC)
bsr7036	2.6	1.7		periplasmic nitrate reductase protein (napE)
blr7037	2.7			periplasmic nitrate reductase (napD)
blr7039	3.2			periplasmic nitrate reductase small subunit precursor (napB)
blr7040	3.3			cytochrome C-type protein (napC)
blr7084	2.1			FNR/CRP-type transcriptional regulator (nnrR)
blr7089	2.6	1.8	1.7	respiratory nitrite reductase (nirK)
blr7090	2.1			probable periplasmic nitrate reductase
blr2804		1.5		nitrate ABC transporter permease protein (nrtB)
**Symbiosis**				
**Nodulation**				
bll1631		1.7		GDP-mannose 4,6-dehydratase (noeL)
blr1632	5.9			putative glucosamine synthase (nodM)
bll2016		2.0		NolY protein (nolY)
blr2024		1.6		NodY protein (nodY)
blr2025		1.9		acyl transferase (nodA)
blr2029		1.8		6-O-carbamoyl transferase (nodU)
blr2034	1.6			NolO protein (nolO)
blr2062		1.6		nodulation protein (noeI)
blr1815	-1.7			nodulation protein (nolV)
bsl2015		-1.6		NolZ protein (nolZ)
bll2019	1.7		-2.4	transcriptional regulatory protein MerR family (nolA)
bll2021			-2.3	transcriptional regulatory protein LysR family (nodD2)
blr2027			-1.5	chitin synthase (nodC)
Heme				
bll1200	2.7	2.0	1.6	5-aminolevulinic acid synthase (EC 2.3.1.37) (hemA)
bll2007	1.8	1.5		coproporphyrinogen III dehydrogenase (hemN1)

bll7086	2.9	2.0	2.6	anaerbic coproporphyrinogen III oxidase (hemN)
blr0469		1.7		heme exporter protein C (ccmC)
**EPS formation**				
bll2362			1.7	succinoglycan biosynthesis transport protein (exoP)
bll4612	-1.5	1.7		putative beta (1-6) glucans synthase (ndvC)
bll7574		1.5		UDP-hexose transferase (exoM)
blr0562		1.8		putative polysaccharide deacetylase
Others				
blr1902	-1.9			putative bacA
**Amino acid biosynthesis**				
bll0244		3.4		probable homoserine O-succinyltransferase (EC 2.3.1.46)
blr0652		1.7		glutamine amidotransferase (hisH)
blr1181		1.8		threonine synthase (thrC)
blr1326		2.0		aspartate ammonium lyase (aspA)
bll1396		1.7		prephenate dehydrogenase (tyrC)
blr1686		1.6		putative aminotransferase protein
blr2371			1.6	serine acetyltransferase (EC 2.3.1.30) (cysE)
bll3765	1.8			glutamine amidotransferase
blr4169		2.1		glutamine synthetase II (glnII)
bll6497		1.6		ketol-acid reductoisomerase (ilvC)
blr7762			1.6	homospermidine synthase
bll7989	1.9			S-adenosylmethionine synthetase (EC 2.5.1.6) (mat)
bll0187		-1.6		3-dehydroquinate synthase (aroB)
blr0488		-1.5		3-isopropylmalate dehydratase large subunit (leuC)
blr0685		-1.9		dihydrodipicolinate reductase (dapB)
blr1323		-1.6		phosphoribosyl-ATP pyrophosphohydrolase (his2)
blr4362	-1.7			homoserine dehydrogenase (hom)
blr5707		-1.5		putative threonine aldolase (EC 4.1.2.-)
bll5902	-1.8	-1.5		threonine dehydratase (ilvA)
bll6039	-1.7			probable SgaA serine-glyoxylate aminotransferase
bll6335	-1.7			similar to ketol-acid reductoisomerase
blr6458		-2.0		glucose-1-phosphate adenylyltransferase (glgC)
blr6687	-1.5			putative phosphoglycerate dehydrogenase
bll7272		-1.7		putative dihydrodipicolinate synthase
bll7969		-1.5		putative dihydrodipicolinate synthase (EC 4.2.1.52)
blr8106		-1.7		succinyl-diaminopimelate desuccinylase (dapE)
**Biosynthesis of cofactors, prosthetic groups, and carriers**				
bll0585		1.6		glutathione S-transferase family protein
bll1380		2.5		thioredoxin-like protein (tlpA)
blr1415		2.0		hypothetical glutathione S-transferase like protein
bll1419		1.7		5,10-methylenetetrahydrofolate reductase (metF)
blr2503	1.7			glutathione S-transferase (gst)
bll4903		1.8		birA bifunctional protein (birA)
bll5026	2.0			H+ translocating pyrophosphate synthase (rrpP)
bll5167	1.8			glutathione S-transferase
blr5775	1.9			putative thioredoxin
blr6542	1.9			hypothetical glutathione S-transferase like protein
blr6659		1.5		thiamine biosynthesis protein (thiC)
bsr6735		1.9	1.8	putative pyrroloquinoline quinone synthesis protein A (pqqA)
blr7480		1.6		riboflavin biosynthesis bifunctional protein (ribF)
blr7556	2.6			non-heme haloperoxidase
bll0532		-1.5		4a-hydroxytetrahydrobiopterin dehydratase

bll1519	-1.9			putative thiamine-phosphate pyrophosphorylase
blr2337		-1.5		4-hydroxybenzoate hydroxylase (pobA)
blr2714		-1.5		ubiquinol oxidase polypeptide II precursor (cyoA)
blr3272	-1.5			cobyrinic acid a,c-diamide synthase (cobB)
blr3276	-1.5			nicotinate-nucleotide--dimethylbenzimidazole phosphoribosyl transferase (cobU)
blr3757		-2.0		glutathione reductase (gor)
blr4233			-1.6	putative pyridoxine kinase (EC 2.7.1.35)
bll5700		-1.7		hypothetical glutathione S-transferase like protein
bsr6656		-1.9		thiamin biosynthesis protein homolog
blr6657		-1.7		thiamine biosynthesis protein (thiG)
bll7452	-1.7			6,7-dimethyl-8-ribityllumazine synthase (ribH)
blr7892		-8.7		putative glutathione S-transferase
**Cell envelope**				
blr1311		1.5		outer membrane protein
blr4700	1.7			putative outer-membrane immunogenic protein precursor
blr4701		1.9		putative outer-membrane immunogenic protein precursor
bll4867	1.7	1.9		putative outer-membrane immunogenic protein precursor
bll5510			1.6	outer-membrane immunogenic protein precursor
bll6888	1.8	1.6		putative porin
bll6948	1.6			putative outer membrane recepter signal peptide protein
bll8196	1.7	1.7		putative outer-membrane immunogenic protein precursor
bll7083			4.8	Putative membrane protein, NnrS-like.
bll7469		-2.9		putative outer-membrane immunogenic protein precursor
bll3035		-2.0		putative outer membrane secretion protein (tolC)
bll4743		-1.8		putative lipoprotein
blr5156		-1.6		putative D-alanyl-D-alanine carboxypeptidase
bll4762		-1.5		putative exopolysaccharide production negative regulator precursor
**Cellular processes**				
**Cell division**				
blr0613		1.5		ammonium transporter (amtB)
blr3970		1.5		cell division protein (ftsJ)
bsr4213			1.7	cell division topological specificity factor (minE)
bll6597		-1.5		cell division protein (ftsA)
bll6598		-2.1		putativecell division protein
**Chaperones**				
blr3152	1.7			Hsp70 family molecular chaperone
blr4635	1.9			chaperonin GroEL
blr4637	2.2			probable HspC2 heat shock protein
bll7789		1.9		probable chaperonine, heat shock hsp90 proteins family (htpG)
blr5234	2.0			small heat shock protein (hspC)
blr5625		1.7		10 KD chaperonin (groES)
blr6571		1.6		small heat shock protein (hspH)
blr6978		1.7		chaperonin (groES2)
blr7961	2.0	1.8		probable HspC2 heat shock protein
bsr7532		1.9		10 KD chaperonin (protein CPN10) (groES)
blr5221	-1.7			small heat shock protein (hspF)
blr5227		-2.0		heat shock protein (groEL1)
blr4653	2.8	-1.6		molecular chaperone, DnaJ family (dnaJ)
bsr3154		-1.6		cold shock protein (cspA)

**Chemotaxis**				
bll1437		1.6		pilus assembly protein (ctpF)
bll1441			1.6	pilus assembly protein prepilin peptidase subunit (ctpB)
bll1532		1.5		putative chemotaxis protein
bll4196		1.9		putative methyl-accepting chemotaxis protein
bll5837		1.5		probable flagellar assembly protein (fliX)
bll5813	1.6			flagellar basal-body rod protein (flgC)
blr5828	1.8			flagellar basal-body rod protein (flgG)
bll6858	1.6			flagellar hook protein (flgE)
bll6882		1.6	1.6	chemotaxis protein (motA)
bll1439		-1.6		pilus assembly protein (ctpD)
bsl1442		-1.7		pilus assembly protein pilin subunit (ctpA)
blr2342		-2.2		chemotaxis two-component response regulator (cheY)
blr2976		-1.6		putative methyl accepting chemotaxis protein
blr3129		-1.9	-2.1	putative methyl accepting chemotaxis protein
bll5309	-1.7			putative methyl-accepting chemotaxis protein
bsl5811	-1.6			flagellar biosynthetic protein (fliQ)
bll5826	-1.5			flagellar synthesis protein (fliL)
bll6874	-1.8			flagellar hook-basal body complex protein (fliE)
bll6878	-1.5			probable flagellar motor switch protein (fliG)
bsl7141		-2.1		components of type IV pilus, pilin subunit (ctpA)
**Protein and peptide secretion**				
bll0641		1.6		protein-export protein (secB)
bll4945			1.6	trigger factor (tig)
bll6222	1.7			probable Sec-independent protein translocase protein
bll6982		2.0		HlyD family secretion protein
blr7872		1.5		HlyD family secretion protein
blr5302		-1.7		HlyD family secretion protein
**Central intermediary metabolism**				
**Hydrogenase**				
blr1737		1.5		hydrogenase expression/formation protein (hypE)
bll6934	1.5			HupJ protein (hupJ)
bll6949	-2.3	-3.6		HupN protein (hupN)
bll6932		-1.9		HypA protein (hypA)
bll6942		-1.5		uptake hydrogenase precursor (hupS)
Others				
bll1011	1.8			sulfur oxidation protein (soxA)
bll1009	1.7			sulfur oxidation protein (soxB)
blr1223	1.5			phosphonate metabolism protein (phnI)
bsl8312		1.9		HNS-type DNA binding protein (hupT)
bll7966		-3.2		putative thiosulfate sulfurtransferase
bll3816		-1.9		putative sulfur-regulated protein
bll5736		-1.8		putative thiosulfate sulfurtransferase precursor (EC 2.8.1.1)
bll7647		-1.5		putative inorganic pyrophosphatase (EC 3.6.1.1)
blr1227		-1.5		phosphonate metabolism protein (phnM)
**DNA replication, recombination, and repair**				
bll0051		1.6		replication protein A (repA)
bll1921		1.8		exonuclease subunit SbcD homolog
bll4601		2.0		transcription-repair coupling factor (mfd)
blr5310	-1.6		1.9	DNA photolyase
blr8051		1.6		putative excinuclease ABC subunit A (uvrA)
blr2499		-1.6		A/G-specific adenine glycosylase (mutY)
blr3165		-2.6		putative Adenine deaminase (EC 3.5.4.2)

bll2509		-1.8		adenine DNA methyltransferase (gst)
blr2963		-1.7		alkylated DNA repair protein (alkB)

Fatty acid, phospholipid and sterol metabolism

bll0225	2.2			acetoacetyl CoA reductase (phbB)
bll0263	1.5			putative malonyl CoA-acyl carrier protein transacylase (EC 2.3.1.39)
blr1288	2.0			probable long-chain-fatty-acid-CoA ligase (EC 6.2.1.3)
blr1309	2.4			acetyl-coenzyme A synthetase (acs)
blr2576	2.5			N-carbamyl-D-amino acid amidohydrolase
blr0509	3.7	1.7		malonyl CoA synthetase
blr2950	3.8			putative enoyl-CoA hydratase (EC 4.2.1.17)
blr3330	7.1	1.6		N-carbamyl-D-amino acid amidohydrolase
blr3414		1.9		putative 4-hydroxybenzoyl CoA thioesterase (EC 3.1.2.23)
bll3752	1.7			molybdenum cofactor biosynthesis protein A (moaA)
blr3956		1.6		enoyl-CoA hydratase
bll7817	3.7			putative acyl-CoA dehydrogenase (EC 1.3.99.-)
bll7818		1.5		putative acyl-CoA dehydrogenase (EC 1.3.99.-)
bll7899		1.8		putative acyl-CoA dehydrogenase (EC 1.3.99.-)
blr0107		-2.3		probable coenzyme A ligase
bll0791		-3.5		apolipoprotein N-acyltransferase (lnt)
blr3329		-1.9		putative long-chain-fatty-acid--CoA ligase (EC 6.2.1.3)
blr3402			-1.6	putative crotonobetaine/carnitine-CoA ligase (EC 6.3.2.-)
blr3537	-1.7			putative carnitinyl-CoA dehydratase (EC 4.2.1.-)
blr3724		-1.6		acetyl-CoA acetyltransferase (phbA)
blr3725	-1.7			acetoacetyl CoA reductase (phbB)
bll3809			-1.8	3-oxoacyl-(acyl-carrier-protein) synthase II (fabF)
bll3810		-4.5	-1.8	hydroxymyristoyl-acyl carrier protein dehydratase
bll3856		-1.6		probable medium-chain-fatty-acid--CoA ligase (EC 6.2.1.-)
blr4085		-1.7		3-oxoacyl-(acyl carrier protein) synthase II (fabF)
bll5944		-2.1		S-adenosylhomocysteine hydrolase (ahcY)
blr6087	-1.5			putative 3-hydroxybutyryl-CoA
bll7820		-1.9		putative long-chain-fatty-acid--CoA ligase (EC 6.2.1.3)
bsl7784		-1.6		probable acyl carrier protein

Purines, pyrimidines, nucleosides, and nucleotides

blr0739	1.5			cytidylate monophosphate kinase (cmk)
blr4088		1.9		guanylate kinase (gmk)
blr6539		1.7		putative adenylate cyclase
bll0758	-1.6			deoxyuridine 5'-triphosphate nucleotidohydrolase (dut)
blr1106	-1.9			2'-deoxycytidine 5'-triphosphate deaminase (dcd)
blr3666		-5.2		nucleoside-diphosphate-sugar epimerase
blr7332		-2.5		putative inosine-5'-monophosphate dehydrogenase protein

Sugars

blr3206	1.6			probable aldose 1-epimerase precursor (EC 5.1.3.3)
bll8129	2.4			UDP-glucose 6-dehydrogenase (ugdH)
bll0323	-1.7			probable trehalose-phosphatase (EC 3.1.3.12) (otsB)
blr0429	-1.5			gamma-glutamyl phosphate reductase (proA)
bll7185	-1.6			probable Glucose 1-dehydrogenase (EC 1.1.1.47) (gdh)
bll6595	-2.1	-1.9		UDP-3-o-(3-hydroxymyristoyl) N-acetylglucosamine deacetylase (lpxC)
blr1120		-1.6		xylose isomerase (xylA)

Respiration

bll0440		1.5		ATP synthase beta chain (atpD)
bll0442		1.8		ATP synthase alpha chain (atpA)

bll1186	-1.6	1.5		FoF1 ATP synthase B' chain (atpB')
bsl1187		2.1		FoF1 ATP synthase C chain (atpC)
blr1248	1.8	1.6		putative thioredoxin reductase
blr3126	1.7	1.5		cytochrome C-type biogenesis protein (cycJ)
blr3728	1.7			cytochrome D ubiquinol oxidase subunit
blr6062	1.5			putative cytochrome C6 precursor
blr6214		2.5	2.0	putative cytochrome c protein
blr6128	2.4			cytochrome c552 (cycB)
blr0151	-2.1			cytochrome O ubiquinol oxidase subunit III (cyoC)
bll0291		-1.6		probable cytochrome B561
bll0557			-1.6	putative cytochrome P450
bll1188	-1.7			FoF1 ATP synthase A chain (atpA)
blr2717	-1.5			cytochrome O ubiquinol oxidase protein (cyoD)
bll2735	-1.6			flavocytochrome C flavoprotein subunit
blr3128		-1.9		cytochrome C-type biogenesis protein (cycL)
bll4483	-4.1			putative cytochrome C4
blr7488	-1.8	-4.9		probable cytochrome C (cyc1)
**Regulatory functions**				
blr0237	6.0			transcriptional regulatory protein LysR family
bll0417		1.5		transcriptional regulatory protein LysR family
blr0675	4.4			heat-inducible transcription repressor (hrcA)
bll0890	1.8			transcriptional regulatory protein Fis family
bll1199			1.6	two-component hybrid sensor and regulator
blr1213		2.0		transcriptional regulatory protein TetR family
bll1220		1.6		transcriptional regulatory protein GntR family
bll2109	2.4			transcriptional regulatory protein Crp family
blr2275		1.5		transcriptional regulatory protein IclR family
blr2331	1.6			transcriptional regulatory protein MarR family
bll2758	1.9	2.0	4.5	two-component response regulator
blr2864		1.6		two-component response regulator
bsl2906	-1.7	1.5		transcriptional regulatory protein TetR family
blr3248	1.8	2.0		transcriptional regulatory protein LysR family
blr3851		1.5		transcriptional regulatory protein MarR family
bll3953		2.3		transcriptional regulatory protein LysR family
blr4002		2.2		transcriptional regulatory protein AraC family
blr4004		1.5		transcriptional regulatory protein ArsR family
blr4195	1.8			transcriptional regulatory protein AraC family
bll4209		1.6		transcriptional regulatory protein TetR family
bll4785	1.9			transcriptional regulatory protein Fis family
blr5120		1.5		transcriptional regulatory protein AraC family
blr5283	1.9			transcriptional regulatory protein IclR family
blr5805	1.9			transcriptional regulatory protein Crp family
bll6060			1.5	putative catabolite gene activator
blr6181		1.7		transcriptional regulatory protein LysR family
bll0330	1.8			two-component response regulator
bll1336	1.5			two-component response regulator
bll6184	2.4			two-component sensor histidine kinase
bll6290		1.5		two-component response regulator
bll6568	4.0			transcriptional regulatory protein LysR family
bll6810		1.9		transcriptional regulatory protein GntR family
bll7479			1.6	two-component response regulator
blr7614	1.6			transcriptional regulatory protein TetR family
blr7666	2.0			transcriptional regulatory protein AraC family
bll7696		2.4		transcriptional regulatory protein Crp family
blr7984	-1.5		2.4	transcriptional regulatory protein TetR family

blr7985			2.4	two-component hybrid sensor and regulator
bsl1986		-1.5		probable transcriptional regulator
bll2628	-1.6			ECF family sigma factor (prtI)
bll2785		-1.5		probable transcriptional regulator
bll6394		-1.7		putative monoamine oxidase regulatory protein
bll1195		-1.8		transcriptional regulator (TtrR)
bll6962	-1.5			transcriptional regulatory protein AraC family
bll2794	-1.6	-2.2		transcriptional regulatory protein AraC family
bll3386		-1.6		transcriptional regulatory protein AraC family
blr4195.1n		-1.6		transcriptional regulatory protein AraC family
bll6252		-1.9		transcriptional regulatory protein AraC family
blr1414		-2.6		transcriptional regulatory protein ArsR family
bll3245	-2.3			transcriptional regulatory protein IclR family
bll3883	-1.5			transcriptional regulatory protein IclR family
blr5288		-1.8		transcriptional regulatory protein IclR family
blr5260	-1.7			transcriptional regulatory protein LysR family
blr7430	-1.7			transcriptional regulatory protein LysR family
bll2504		-1.5		transcriptional regulatory protein LysR family
blr5093		-1.5		transcriptional regulatory protein LysR family
blr5870			-1.7	transcriptional regulatory protein LysR family
bll2260	-2.4			transcriptional regulatory protein MarR family
bll4381	-1.5			transcriptional regulatory protein MarR family
blr2685		-2.0		transcriptional regulatory protein MarR family
blr6889	-2.0			transcriptional regulatory protein MarR family
blr2971		-1.7		transcriptional regulatory protein MarR family
bll5036		-2.3		transcriptional regulatory protein MarR family (mucS)
bll4010	-1.5			transcriptional regulatory protein PadR-like family
bll7139	-1.6			transcriptional regulatory protein TetR family
blr3838		-2.4		transcriptional regulatory protein TetR family
bll6143		-3.7		transcriptional regulatory protein TetR family
bll5886		-3.2		two-component hybrid sensor and regulator
bll0391		-53.2		two-component response regulator
blr2285	-1.5			two-component response regulator
blr5281	-2.0			two-component response regulator
blr6651		-1.6		two-component response regulator
bll7342		-1.7		two-component response regulator
bll7658		-1.5		two-component response regulator
bll7795	-2.3			two-component response regulator
blr3122		-1.9		two-component sensor histidine kinase
bll0905		-3.1		two-component sensor histidine kinase (regS)
**Transcription**				
bll0458	-1.5			2'-5' RNA ligase (ligT)
bll0779		1.8	1.6	polyribonucleotide nucleotidyltransferase (pnpA)
bll1447		2.0		dead-box ATP-dependent RNA helicase (rhlE)
bll4348		1.7		ATP-dependent RNA helicase (rhlE)
blr4117		-1.5		putative nuclease
blr7797	-1.7	-1.9		RNA polymerase sigma-E factor (Sigma-24) protein
blr4928		-1.9		ECF family sigma factor (SigD)
blr7337		-1.7		sigma32-like transcription factor (rpoH2)
blr2557		-1.6		putative RNA polymerase sigma factor protein
blr2203		-1.5		putative RNA polymerase sigma factor
**Translation**				
**Ribosomal proteins**				
blr0482		1.7		30S ribosomal protein S16 (rpsP)

bll4076		1.6		50S ribosomal protein L9 (rplI)
bll4079		1.9		30S ribosomal protein S6 (rpsF)
bll4861		2.7		30S ribosomal protein S2
bll4963		1.5		50S ribosomal protein L13 (rplM)
bll5377		1.7	1.8	30S ribosomal protein S11 (rpsK)
bll5378		1.5		30S ribosomal protein S13 (rpsM)
bsl5382			1.6	50S ribosomal protein L30 (rpmD)
bll5383		1.6	1.9	30s ribosomal protein S5 (rpsE)
bll5384		1.7		50S ribosomal protein L18 (rplR)
bsl5391		2.0	1.7	30S ribosomal protein S17 (rpsQ)
bsl5392		1.7		50S ribosomal protein L29 (rpmC)
bll5393		1.7		50S ribosomal protein L16 (rplP)
bll5394		1.9		30S ribosomal protein S3 (rpsC)
bsl5396		1.8		30S ribosomal protein S19 (rpsS)
bll5399		1.9		50S ribosomal protein L4 (rplD)
bll5400		1.7		50S ribosomal protein L3 (rplC)
bll5401	-1.8	1.6		30S ribosomal protein S10 (rpsJ)
bll5405	1.8	2.1		30S ribosomal protein S12 (rpsL)
bll5411		1.8		50S ribosomal protein L7/L12 (rplL)
bll5412		2.0		50S ribosomal Protein L10 (rplJ)
bll7441		1.5		50S ribosomal protein L25 (rplY)
bsr5117	-2.1			50S ribosomal protein L33 (rpmG)
bsr7117	-1.5			30S ribosomal protein S21 (rpsU)
bsr8096	-1.6			ribosomal protein L34
**Degradation of proteins, peptides, and glycopeptides**				
blr1404	1.9	1.5		ATP-dependent protease, ATP-binding subunit (clpB)
bll4639	1.7			probable protease
Others				
bll4403.1n	7.9			proline iminopeptidase
bll1795		1.9		similar to zinc protease
blr2591		1.8		serine protease DO-like protease (dop)
bll4854	3.1	3.2		zinc metallopeptidase
bll5267		1.6		propionyl-coenzyme A carboxylase, alpha polypeptide (pccA)
blr5311		1.7		probable histon H1
bll5402		2.2	1.7	elongation factor TU (tuf)
bll5403		2.0		translation elongation factor G (fusA)
blr7274	1.5			serine protease
bll7440		1.7	1.5	peptidyl-tRNA hydrolase (EC 3.1.1.29) (pth)
bll0781	-1.7			tRNA pseudouridine 55 synthase (truB)
bll6260	-1.5			peptide methionine sulfoxide reductase
**Transport and binding proteins**				
bll0089	1.9			ABC transporter permease protein
bll0197	2.9			ABC transporter permease protein
bll0193		2.7		ABC transporter ATP-binding protein
blr0998	2.1			probable periplasmic substrate binding protein
bll1023			1.5	probable amino acid binding protein
blr1017	1.7			MFS permease
blr1354			1.9	peptide ABC transporter permease protein
blr1355		2.9	2.0	peptide ABC transporter permease protein
blr1448			1.5	ABC transporter substrate-binding protein
blr1452		2.0		ABC transporter ATP-binding protein
bll2304	1.5			probable ABC transporter, substrate binding protein

blr2314		1.7		MFS permease
blr2422	1.6			putative efflux protein
blr2439	1.6			ABC transporter permease protein
blr2440		1.9		ABC transporter permease protein
blr2442		2.2		ABC transporter substrate-binding protein
bll2731		1.5		probable ABC transporter substrate-binding protein
blr2934		1.5		probable cation efflux protein (ragC)
blr3183	1.7			ABC transporter substrate-binding protein
blr3209		1.6		sugar ABC transporter ATP-binding protein
blr3344		1.5		ABC transporter ATP-binding protein
bll3652		1.5		probable O-antigen export system permease protein (rfbD)
bll3708	2.9			efflux protein
blr3723	1.6			C4-dicarboxylate transport protein (dctA)
blr3815	2.2			putative cation-transporting ATPase (EC 3.6.3.-)
bll3875		1.7		hypothetical metabolite transport protein
bll3971	1.8			MFS permease
blr4112	2.7			Probale cation efflux system protein
blr4115	1.7	1.5		putative symporter
bll4204		2.2		probable ATP-binding protein
blr4449	1.8			amino acid ABC transporter ATP-binding protein
bsr4636	3.6			putative cation transport regulator
bll5056		1.6		probable substrate-binding protein
blr5576	1.9			ABC transporter permease protein
bll5771	1.9			AcrB/AcrD/AcrF family cation efflux protein
bll5080	1.6			AcrB/AcrD/AcrF family protein
blr5724	2.7			probable metal-transport protein
bll6063	1.6		1.6	ABC transporter substrate-binding protein
bll6064	1.6			ABC transporter ATP-binding protein
bll6065	1.6			ABC transporter permease protein
blr6156		4.3		ABC transporter permease protein
blr6157	5.9			ABC transporter permease protein
bll6389		1.7		ABC transporter permease protein
bll6406	1.7			ABC transporter ATP-binding protein
bll6450		1.7		probable substrate-binding protein
bll6481	2.0			ABC transporter substrate-binding protein
bll6487		1.9		ABC transporter permease protein
bll6685			1.5	probable ATP-binding protein
bll7008	1.5			aliphatic sulfonate ABC transporter ATP-binding protein
blr7053	2.1			probable cation-transporting ATPase (EC 3.6.3.-)
bll7103	2.1			ABC transporter permease protein
bll7988	1.9			probable ATP-binding protein
blr8118		2.2		ABC transporter permease protein
bll0733		-2.3		ABC transporter glycerol-3-phosphate-binding protein
bll0381	-1.5	-1.6		ABC transporter substrate-binding protein
bll0889	-1.6			putative transport protein
blr1039		-4.3		ABC transporter ATP-binding protein
blr1066			-2.0	ABC transporter ATP-binding protein
blr1067	-1.8	-9.8	-2.2	ABC transporter ATP-binding protein
blr1451		-1.9		ABC transporter ATP-binding protein
bll2870	-1.5			ABC transporter ATP-binding protein
blr3099			-1.6	ABC transporter ATP-binding protein
blr3186			-1.5	ABC transporter ATP-binding protein
blr3345		-1.5		ABC transporter ATP-binding protein
bll4892	-1.8			ABC transporter ATP-binding protein
bll5649		-1.6		ABC transporter ATP-binding protein

bll5950		-1.6		ABC transporter ATP-binding protein
bll6368		-2.7		ABC transporter ATP-binding protein
blr6447	-1.9			ABC transporter ATP-binding protein
bll6477		-2.1		ABC transporter ATP-binding protein
blr7933	-1.9			ABC transporter ATP-binding protein
blr2753		-1.6		ABC transporter HlyB/MsbA family
blr3795	-1.7	-2.6		ABC transporter HlyB/MsbA family
blr1065		-1.9		ABC transporter permease protein
blr3185			-1.7	ABC transporter permease protein
bll3314	-3.8			ABC transporter permease protein
bll6020	-1.5			probable general secretion pathway protein F
blr6150	-1.6			ABC transporter permease protein
bll6403		-1.9		ABC transporter permease protein
bll6824		-1.6		ABC transporter permease protein
bll7770		-1.7		ABC transporter permease protein
bll2868	-1.7			ABC transporter substrate-binding protein
bll3192	-1.7			ABC transporter substrate-binding protein
bll3316		-2.0		ABC transporter substrate-binding protein
blr3547	-1.7			ABC transporter substrate-binding protein
bll4896		-1.8		ABC transporter substrate-binding protein
blr5675	-2.6			ABC transporter substrate-binding protein
bll7921		-1.8		ABC transporter substrate-binding protein
bll3962	-1.5			MFS permease
bll5121	-1.7			major facilitator superfamily transporter
bll1394		-1.9		cation transport protein (chaC)
bll3948		-1.6		monocarboxylate MFS permease
blr3743		-1.6		periplasmic mannitol-binding protein
blr1225		-1.9		phosphonate uptake transporter ATP-binding protein (phnK)
bll4878		-1.6		possible Copper export protein
blr1052	-2.0			putative ABC transporter binding protein
blr2912		-1.9		probable ABC transporter permease protein
blr4500		-1.9		probable ABC transporter permease protein
blr7066		-1.5		probable ABC transporter permease protein
blr4555	-1.9			similar to ABC transporter permease protein
bll6709		-1.5		probable ATP-binding protein
blr7537		-1.5		probable ATP-binding protein
blr5727		-1.5		probable metal-transport protein
blr2848		-1.6		probable sulphate transport protein
blr7376		-1.6		putative integral membrane transporter protein
bll0324		-1.5		putative sugar transport protein
blr6555		-1.7		putative sulfate transporter
bll5251		-1.8		putative transporter transmembrane protein
blr8132		-1.5		RhtB family transporter
blr1121		-1.6		sugar ABC transporter ATP-binding protein
blr6569		-1.6		MFS permease
bll5620		-1.5		MFS permease
blr3904	-1.5			probable iron transport protein

Other categories

Drug and analog sensitivity

blr2617	1.8			metallo-beta-lactamase superfamily protein
bll7407			2.0	fosmidomycin resistance protein (fsr)
blr7593	1.8	2.0		multidrug resistance efflux pump
blr4439		6.9	2.6	penicillin binding protein
blr7958	-1.5	-1.7		multidrug resistance efflux pump

bll5081		-1.6		putative multidrag resistance protein
**Transposon-related functions**				
blr0018.1			1.8	putative transposase
blr0018.2		1.7	1.8	putative transposase
bll1642		1.9		putative transposase
blr1645	2.0			putative transposase
blr1655		1.8		putative transposase
blr1740	1.6			RSalpha~putative transposase
bll1742		1.6		putative transposase
bsr1874		1.6		putative transposase
blr2056		1.6		putative transposase
bll2116			1.8	putative transposase
bll2117			1.5	putative transposase
bll4642	2.1			putative transposase
blr8004		15.5		probable site-specific integrase/recombinase
bll8183	1.6			putative transposase
blr8217		1.5		putative transposase
blr1717	-1.6			putative transposase
bll1997	-1.5			putative transposase
bll2160		-5.2		putative transposase
blr5169	-1.6			probable site-specific integrase/recombinase
bll8182		-2.1		putative transposase
bll8199		-1.9		putative transposase
blr8301	-1.7			putative transposase
blr1617	-1.9			conjugal transfer protein (trbL)
Others				
bll0100	1.7			ferredoxin NADP+ reductase
bll0134	2.3			putative oxidoreductase
bll0137	2.5			hypothetical oxidoreductase
bll0409	1.7			probable FAD-monooxygenase
bll0527	1.6			hypothetical oxidoreductase
blr0568	1.8			glycerol-3-phosphate dehydrogenase [NAD(P)+] (EC 1.1.1.94) (gpsA)
blr0573	1.9			acetyl-CoA synthetase
bll1234	2.1			putative hydrolase
bll1503	1.9			quinone oxidoreductase (qor >
bll2377		1.6		probable glycosyl transferase
bll2517	1.6			putative acetate kinase (acetokinase) protein
bll2569	1.6			FAD dependent oxidoreductase
bll3020		1.5		GMC type oxidoreductase
bll3142		1.5		putative quinone oxidoreductase (EC 1.6.5.5)
bll3147		2.0		putative 2-dehydropantoate 2-reductase (EC 1.1.1.169)
blr3457	2.0			phosphate acetyltransferase (EC 2.3.1.8) (pta)
blr3776	2.0			betaine aldehyde dehydrogenase
bll4001		1.9		putative esterase
blr4257	1.6			putative hydrolase
bll4301		1.6		putative oxidoreductase protein
bll4346		1.7		3-hydroxydecanoyl-[acyl-carrier-protein] dehydratase (EC 4.2.1.60) (fabA)
bll4493	1.7			putative acetyltransferase (EC 2.3.1.-)
blr4659	1.5			PfkB family carbohydrate kinase
blr5498	1.6			putative oxidoreductase protein
bll5683	1.6			probable serine/threonine phosphatase
blr5774	1.9			probable sulfide-quinone reductase
blr6059	1.7			putative cyclase

bll6073	1.6			probable poly-beta-hydroxybutyrate polymerase (phbC)
bll6180	1.7	1.7		oxidoreductase
blr6671	1.9			dehydrogenase
bll6826	1.9			putative oxidoreductase
bll6994		2.2		putative phosphatidylethanolamine N-methyltransferase
blr7063	1.7			probable 2-ketogluconate reductase
bll7207			1.5	probable N-Carbamoyl-D-Amino-Acid
blr7884		1.7		probable coniferyl aldehyde dehydrogenase (calB)
bll7886	2.7			probable 3-oxoacyl-[acyl-carrier-protein] reductase
bll8067	-1.6	3.3		similar to LpqO protein
bll8163		2.0		glycosyl transferase (gtrA)
blr0899		1.5		poly(3-hydroxyalkanoate) depolymerase
blr2168		1.8		putative transketolase alpha subunit protein
blr2459	1.5	2.6		acetyltransferase
blr2916	1.6			tartrate dehydrogenase
bll3116	1.9			putative phosphoribosylpyrophosphate synthetase
blr3320		1.6		hypothetical oxidoreductase
blr3335		5.2		3-oxoacyl-[acyl-carrier-protein] reductase
blr3449	1.8			probable dehydrogenase
blr4420		1.7		3-methylcrotonoyl-CoA carboxylase beta subunit (mccB)
blr6661		1.8		putative glycosylase
blr6744		2.8		putative ferredoxin oxidoreductase beta subunit
blr6983		1.6		probable 1,4-butanediol diacrylate esterase
blr7262		1.7		putative racemase
blr7422		2.7		oxidoreductase
blr7568	1.9			flavoprotein (wrbA)
blr7602		2.3		putative monooxygenase
bsr6522		1.5		Putative FeoA family protein
bll2208		5.0		putative periplasmic copper binding protein
bll0043			3.1	conjugal transfer protein (traG)
bll8285		1.5		probable conjugal transfer protein (trbE)
bll0290	-1.6			hypothetical low temperature-induced protein
bll0554			-1.6	short-chain dehydrogenase
bll0566		-1.6		putative uroporphyrinogen III synthase
bll0766	-1.8			probable dehydrogenase
bll1230		-1.6		3-oxoacyl-[acyl-carrier-protein] reductase
bll1531	-1.5			probable oxidoreductase
bll1586	-1.8			putative excisionase
blr2102	-1.7			pantoate—beta-alanine ligase (panC)
bll2434	-3.3			plasmid stability protein
bll2527		-1.7		putative hydrolase
bll2752		-1.7		probable glycosyl transferase
blr2810	-1.5			aldo/keto reductase
bll2850			-1.5	probable 6-phosphofructokinase (EC 2.7.1.11)
bll2927		-1.5		putative aldolase, possibly L-fuculose-phosphate aldolase
bll3065		-2.3		hypothetical aldolase class II protein
bll3109	-1.7			putative Ca binding protein
bll3238	-1.6			hydantoinase A (hyuA)
bll3418	-2.0	-1.9		putative epoxide hydrolase 1 (EC 3.3.2.3)
bll3658		-1.7		putative dehydrogenase
bll4164		-3.5		putative oxidoreductase
bll4669	-1.7			putative mutator protein
bll4688	-1.5			putative acetyltransferase (EC 2.3.1.-)
bll5049	-2.0	-1.6		AttM/AiiB family protein
bll5282		-2.4		biphenyl-2,3-diol 1,2-dioxygenase

bll5335	-1.8			putative thiolase
bll5831	-1.7			putative hydratase (EC 4.2.1.-)
bll6323		-1.7		putative 3-hydroxyisobutyrate dehydrogenase (EC 1.1.1.31)
bll6359	-1.6			putative oxidoreductase protein
bll6610		-1.5		S-adenosyl-methyltransferase (mraW)
bll7774	-1.6			superoxide dismutase (sodF)
bll8141	1.7	-1.6		phosphoenolpyruvate carboxykinase (pckA)
bll8165	-1.6			putative alkaline phosphatase protein
blr0463		-3.1		acetyltransferase
blr0579		-1.6		SUN protein (sun)
blr0589		-1.6		probable 2-dehydropantoate 2-reductase (EC 1.1.1.169)
blr2174		-1.5		malonic semialdehyde oxidative decarboxylase (iolD)
blr2281		-1.5		putative 2-pyrone-4,6-dicarbaxylate hydrolase
blr2938		-2.7		cyclohexanone monooxygenase
blr3218		-1.5		putative hydrolase phosphatase protein
blr3336	-1.6			oxidoreductase
blr3400	-1.7			putative oxidoreductase
blr3401		-1.6		probable aromatic 1,2-dioxygenase beta subunit
blr3403		-4.5	-1.6	putative 3-oxoacyl-[acyl-carrier-protein] reductase
blr3672		-1.6		oxidoreductase
blr3677	-1.8	-1.7		putative monooxygenase component
blr3679	-1.6			putative monooxygenase component
blr3799		-1.7		probable oxidoreductase
blr3819		-1.5		metapyrocatechase
blr4190		-1.5		putative oxidoreductase
blr4217		-1.7		putative oxidoreductase
blr4404	-1.7			histone-like protein
blr5116		-1.5		Nrd protein (nrd)
blr5209		-1.6		oxidoreductase
blr5371	-1.8			zinc-binding dehydrogenase
blr5421	-1.6			dehydratase-like protein
blr5444		-1.7		putative oxidoreductase protein
blr5591	-1.5			salicylate hydroxylase (nah)
blr5658	1.6		-1.5	putative avidin
blr5871		-3.9		5-dehydro-4-deoxyglucarate dehydratase
blr6721			-1.5	putative reductase
blr6094	-1.6			oxidoreductase
blr6161	-1.7			putative dehydrogenase
blr7165	-1.7	-1.9		oxidoreductase
blr7491	-1.7			putative 2-keto-gluconate dehydrogenase
bll7539	-2.1			probable histone H1
**Hypothetical proteins**				
bsr0004		1.7		hypothetical protein bsr0004
blr0035		5.9		hypothetical protein blr0035
bll0042	-1.5		2.9	hypothetical protein bll0042
bll0044		2.7		hypothetical protein bll0044
bll0048		1.7		hypothetical protein bll0048
bll0052	2.0	3.2		hypothetical protein bll0052
bsr0067		2.0		hypothetical protein bsr0067
bll0083		1.6		hypothetical protein bll0083
blr0094	2.3			hypothetical protein blr0094
bsl0098		1.9		hypothetical protein bsl0098
blr0123	1.5			hypothetical protein blr0123
bll0177		1.9		hypothetical protein bll0177
bsl0255	1.7			hypothetical protein bsl0255

bsl0296.2n	1.5			hypothetical protein bsl0296.2n
blr0349	2.0			hypothetical protein blr0349
bll0491	1.8			hypothetical protein bll0491
blr0496			2.6	hypothetical protein blr0496
blr0497	1.8	3.5		hypothetical protein blr0497
blr0538.1n		1.7		hypothetical protein blr0538
blr0569		1.7		hypothetical protein blr0569
bll0596	1.5			hypothetical protein bll0596
blr0629	1.7	1.6		hypothetical protein blr0629
blr0644		1.8		hypothetical protein blr0644
bll0657		1.6		hypothetical protein bll0657
blr0727		3.8		hypothetical protein blr0727
bsl0728	1.5			hypothetical protein bsl0728
blr0743	1.5			hypothetical protein blr0743
bll0786	2.6			hypothetical protein bll0786
bll0793		1.6		hypothetical protein bll0793
bsl0808	2.0			hypothetical protein bsl0808
bll0828	1.7	6.9		hypothetical protein bll0828
bll0831		1.7		hypothetical protein bll0831
blr0850		6.2	1.5	hypothetical protein blr0850
blr0860		2.2		hypothetical protein blr0860
bll0880	1.6	1.5		hypothetical protein bll0880
blr0908	1.6			hypothetical protein blr0908
blr0920		1.5		hypothetical protein blr0920
bsl1006	1.9			hypothetical protein bsl1006
bsl1061		1.5		hypothetical protein bsl1061
blr1079			1.6	hypothetical protein blr1079
blr1125		2.3		hypothetical protein blr1125
blr1147	7.3	2.0		hypothetical protein blr1147
bll1153		2.7		hypothetical protein bll1153
blr1178		4.8		hypothetical protein blr1178
blr1247		1.6		hypothetical protein blr1247
bll1287		1.5		hypothetical protein bll1287
blr1289	2.4	2.7		hypothetical protein blr1289
blr1349.2n	1.6			hypothetical protein blr1349.2n
blr1402		1.6		hypothetical protein blr1402
bsr1553	3.5	2.8		hypothetical protein bsr1553
blr1563	1.8			hypothetical protein blr1563
blr1611		1.8		hypothetical protein blr1611
blr1649.5n		1.8		hypothetical protein blr1649
blr1665	1.6			hypothetical protein blr1665
blr1693			1.7	hypothetical protein blr1693
blr1716.2	2.0			hypothetical protein blr1716.2
bsl1845		2.0		hypothetical protein bsl1845
blr1905	1.9			hypothetical protein blr1905
blr1945	-1.6	1.7		hypothetical protein blr1945
blr1998		6.1		hypothetical protein blr1998
blr2044	1.8			hypothetical protein blr2044
bsl2064			1.7	hypothetical protein bsl2064
bsl2070	2.1		2.0	hypothetical protein bsl2070
bsr2111		2.0		hypothetical protein bsr2111
blr2232		1.8		hypothetical protein blr2232
blr2279	1.8			hypothetical protein blr2279
bll2302	1.8	1.7		hypothetical protein bll2302
bsl2328	3.4			hypothetical protein bsl2328
bll2330	1.8			hypothetical protein bll2330

blr2372			1.5	hypothetical protein blr2372
blr2409		1.9		hypothetical protein blr2409
bll2431		2.7		hypothetical protein bll2431
bll2446	3.6			hypothetical protein bll2446
blr2466		1.9		hypothetical protein blr2466
blr2470.1n	1.8			hypothetical protein blr2470.1n
bll2471	2.0	1.9		hypothetical protein bll2471
blr2488	1.9			hypothetical protein blr2488
blr2505		1.5		hypothetical protein blr2505
bll2516	1.5			hypothetical protein bll2516
bll2518	2.6			hypothetical protein bll2518
blr2544	2.0			hypothetical protein blr2544
bll2561	2.4			hypothetical protein bll2561
bsl2575	1.5			hypothetical protein bsl2575
bll2590	2.0	2.4	1.6	hypothetical protein bll2590
bsl2602.1n	1.7			hypothetical protein bsl2602.1n
blr2611		1.9		hypothetical protein blr2611
bll2645	2.6			hypothetical protein bll2645
bll2647	2.0			hypothetical protein bll2647
blr2659	2.9			hypothetical protein blr2659
bll2662	2.2			hypothetical protein bll2662
bll2663	2.9			hypothetical protein bll2663
blr2666	2.1			hypothetical protein blr2666
bsr2670	1.6			hypothetical protein bsr2670
blr2671	1.8			hypothetical protein blr2671
bsr2672	2.4			hypothetical protein bsr2672
bsl2673		1.5		hypothetical protein bsl2673
bll2704		1.7		hypothetical protein bll2704
bll2708		1.6		hypothetical protein bll2708
blr2726		2.0		hypothetical protein blr2726
blr2761	2.1	2.0		hypothetical protein blr2761
blr2762	2.3	1.9		hypothetical protein blr2762
bsr2788		1.8		hypothetical protein bsr2788
bll2784	2.5			hypothetical protein bll2784
blr2818	1.6			hypothetical protein blr2818
blr2842		2.7		hypothetical protein blr2842
bsr2847	1.8			hypothetical protein bsr2847
blr2854	1.5			hypothetical protein blr2854
blr2865	1.7			hypothetical protein blr2865
blr2930		2.0		hypothetical protein blr2930
blr2975	1.8			hypothetical protein blr2975
blr2987	1.7			hypothetical protein blr2987
bll3000		1.6		hypothetical protein bll3000
blr3001		2.3		hypothetical protein blr3001
bll3019		1.9		hypothetical protein bll3019
blr3025	1.9			hypothetical protein blr3025
bll3051	2.0	2.4		hypothetical protein bll3051
bll3115	2.9			hypothetical protein bll3115
bll3153		1.7		hypothetical protein bll3153
bsr3236	1.7			hypothetical protein bsr3236
bll3246	1.6			hypothetical protein bll3246
bll3300		1.8		hypothetical protein bll3300
bll3359		1.7		hypothetical protein bll3359
blr3407	1.6			hypothetical protein blr3407
blr3455	1.6			hypothetical protein blr3455
blr3456	2.2			hypothetical protein blr3456

blr3478	1.8			hypothetical protein blr3478
bll3480	1.5			hypothetical protein bll3480
bll3481	1.9	1.5		hypothetical protein bll3481
blr3486	1.5			hypothetical protein blr3486
blr3487	2.7			hypothetical protein blr3487
bll3509	1.9			hypothetical protein bll3509
bll3645	4.2	2.5		hypothetical protein bll3645
bsl3687		3.2		hypothetical protein bsl3687
bll3690		1.5		hypothetical protein bll3690
bsl3746	1.5			hypothetical protein bsl3746
bll3753			1.7	hypothetical protein bll3753
bll3759.1n		2.4		hypothetical protein bll3759
blr3767	2.3			hypothetical protein blr3767
bll3794		2.0		hypothetical protein bll3794
blr3861		2.2		hypothetical protein blr3861
bsr3925	1.9	1.5		hypothetical protein bsr3925
bll4008	1.5			hypothetical protein bll4008
blr4029	1.6			hypothetical protein blr4029
bll4032		1.6		hypothetical protein bll4032
blr4050	2.0			hypothetical protein blr4050
bll4089			1.7	hypothetical protein bll4089
blr4111	2.1			hypothetical protein blr4111
blr4113	2.0			hypothetical protein blr4113
blr4114	1.9	2.1		hypothetical protein blr4114
bsl4153		1.5		hypothetical protein bsl4153
blr4155		1.5		hypothetical protein blr4155
blr4174	1.8			hypothetical protein blr4174
bsr4175	1.6			hypothetical protein bsr4175
blr4188		1.5		hypothetical protein blr4188
blr4199		1.6		hypothetical protein blr4199
bll4208		1.7		hypothetical protein bll4208
bll4220		1.5		hypothetical protein bll4220
bsr4225			1.8	hypothetical protein bsr4225
blr4238			3.2	hypothetical protein blr4238
bll4239	1.5			hypothetical protein bll4239
blr4241	1.8	1.9		hypothetical protein blr4241
bll4269	1.8			hypothetical protein bll4269
bll4329		1.5		hypothetical protein bll4329
bll4336		1.5		hypothetical protein bll4336
blr4350		2.0		hypothetical protein blr4350
bll4354		1.8		hypothetical protein bll4354
bll4412	2.4	3.8		hypothetical protein bll4412
blr4438			2.4	hypothetical protein blr4438
blr4452		1.8		hypothetical protein blr4452
blr4454	6.5			hypothetical protein blr4454
blr4470		1.7		hypothetical protein blr4470
blr4495		1.5		hypothetical protein blr4495
blr4507	1.6			hypothetical protein blr4507
bsl4527		1.6		hypothetical protein bsl4527
bsl4534		1.7		hypothetical protein bsl4534
blr4563	1.7			hypothetical protein blr4563
bsl4569	3.9			hypothetical protein bsl4569
bsl4602	1.8			hypothetical protein bsl4602
blr4633	1.5			hypothetical protein blr4633
bll4634	1.6			hypothetical protein bll4634
blr4638	2.6			hypothetical protein blr4638

bll4640	2.2			hypothetical protein bll4640
blr4641	2.3			hypothetical protein blr4641
bll4643	2.6		2.3	hypothetical protein bll4643
bll4644	2.7			hypothetical protein bll4644
bll4645	2.3			hypothetical protein bll4645
blr4646	2.6			hypothetical protein blr4646
bsl4647	1.8			hypothetical protein bsl4647
bsl4650	2.7			hypothetical protein bsl4650
bll4651	3.6			hypothetical protein bll4651
blr4652	2.7			hypothetical protein blr4652
blr4654	3.1	2.2		hypothetical protein blr4654
bll4706			1.5	hypothetical protein bll4706
bll4707		1.7		hypothetical protein bll4707
bll4771		2.0		hypothetical protein bll4771
bll4787		1.7		hypothetical protein bll4787
bll4799		1.6		hypothetical protein bll4799
bll4817	2.7			hypothetical protein bll4817
bll4818	1.6			hypothetical protein bll4818
bsr4821	2.0			hypothetical protein bsr4821
blr4827	1.5			hypothetical protein blr4827
bll4869	1.6			hypothetical protein bll4869
blr4890	1.6			hypothetical protein blr4890
bll4907			1.5	hypothetical protein bll4907
bll4941		3.0		hypothetical protein bll4941
bsr4956	2.1			hypothetical protein bsr4956
bsr4957	1.8			hypothetical protein bsr4957
blr4982		1.5		hypothetical protein blr4982
bll4983	1.6			hypothetical protein bll4983
blr4994	4.5	1.7		hypothetical protein blr4994
bll5001	1.6			hypothetical protein bll5001
bsl5002	2.4			hypothetical protein bsl5002
blr5025		1.6		hypothetical protein blr5025
bsr5054		9.8		hypothetical protein bsr5054
bll5076	2.5			hypothetical protein bll5076
bll5077		1.6		hypothetical protein bll5077
bll5078		1.7		hypothetical protein bll5078
bll5079	2.1			hypothetical protein bll5079
bsl5086		1.5		hypothetical protein bsl5086
blr5113		1.7		hypothetical protein blr5113
blr5140	1.6			hypothetical protein blr5140
blr5150	2.5			hypothetical protein blr5150
blr5174		1.7		hypothetical protein blr5174
bll5191	3.7			hypothetical protein bll5191
bll5196		1.6		hypothetical protein bll5196
bll5202	2.9			hypothetical protein bll5202
bll5205	1.6			hypothetical protein bll5205
bsr5206		1.9		hypothetical protein bsr5206
blr5222	1.5			hypothetical protein blr5222
bsr5223			1.6	hypothetical protein bsr5223
bsl5224		1.6		hypothetical protein bsl5224
blr5428	2.0			hypothetical protein blr5428
blr5431	2.4			hypothetical protein blr5431
bll5241		1.5		hypothetical protein bll5241
blr5258		1.6		hypothetical protein blr5258
bll5254		1.8		hypothetical protein bll5254
bsr5273	2.2		2.5	hypothetical protein bsr5273

bll5314		4.4	1.8	hypothetical protein bll5314
bll5315	2.7		4.1	hypothetical protein bll5315
bll5315.1n		1.5	2.2	hypothetical protein bll5315
bll5331		1.9		hypothetical protein bll5331
blr5435		1.6		hypothetical protein blr5435
blr5540		1.6		hypothetical protein blr5540
bsr5463		1.7		hypothetical protein bsr5463
bll5495		1.6		hypothetical protein bll5495
blr5636		2.1		hypothetical protein blr5636
bll5660		2.2		hypothetical protein bll5660
bll5663			2.2	hypothetical protein bll5663
bll5696		1.5		hypothetical protein bll5696
bll5754		1.9		hypothetical protein bll5754
bsr5760		1.6		hypothetical protein bsr5760
bll5766			2.0	hypothetical protein bll5766
bll5770	2.1		1.8	hypothetical protein bll5770
bll5772	2.6			hypothetical protein bll5772
bsr5776	2.4			hypothetical protein bsr5776
blr5777	2.3			hypothetical protein blr5777
bll5851		1.8		hypothetical protein bll5851
bll5984	2.0			hypothetical protein bll5984
blr6042	1.6			hypothetical protein blr6042
bll6051		1.7		hypothetical protein bll6051
bsr6066	2.8	2.2		hypothetical protein bsr6066
blr6067	2.0			hypothetical protein blr6067
bll6068	2.1			hypothetical protein bll6068
bll6069	1.8		1.8	hypothetical protein bll6069
blr6071	2.4			hypothetical protein blr6071
blr6074	1.6	2.9		hypothetical protein blr6074
bll6075	1.6			hypothetical protein bll6075
bll6179	1.5			hypothetical protein bll6179
blr6196	2.0			hypothetical protein blr6196
bll6205		1.9		hypothetical protein bll6205
bsl6345		1.6		hypothetical protein bsl6345
bll6375		1.5		hypothetical protein bll6375
bll6422	1.8			hypothetical protein bll6422
bsr6466			2.4	hypothetical protein bsr6466
bll6483		1.9		hypothetical protein bll6483
blr6518		2.2		hypothetical protein blr6518
bsr6520		2.1	5.4	hypothetical protein bsr6520
bsr6521	1.8	2.1	2.4	hypothetical protein bsr6521
bsr6522	2.0			hypothetical protein bsr6522
blr6541	1.5			hypothetical protein blr6541
blr6544	1.7			hypothetical protein blr6544
bll6583	6.3			hypothetical protein bll6583
bll6673	2.2			hypothetical protein bll6673
blr6682		1.7		hypothetical protein blr6682
bll6893	1.6			hypothetical protein bll6893
bsr6700			2.1	hypothetical protein bsr6700
bll7022		1.5		hypothetical protein bll7022
blr7054	1.8			hypothetical protein blr7054
bll7056		1.6		hypothetical protein bll7056
blr7060	2.9			hypothetical protein blr7060
blr7069		1.5		hypothetical protein blr7069
bsr7087	1.7			hypothetical protein bsr7087
blr7088	2.2		1.8	hypothetical protein blr7088

bll7134	2.5			hypothetical protein bll7134
bll7119			1.6	hypothetical protein bll7119
bll7160	1.8	2.1		hypothetical protein bll7160
bll7183.1n		1.6		hypothetical protein bll7183
bll7210	1.5			hypothetical protein bll7210
bll7211		1.9		hypothetical protein bll7211
bll7225			2.5	hypothetical protein bll7225
bll7226			2.5	hypothetical protein bll7226
blr7300		2.8		hypothetical protein blr7300
blr7314		2.4		hypothetical protein blr7314
blr7345	2.2			hypothetical protein blr7345
bll7353	2.4			hypothetical protein bll7353
blr7360	1.8			hypothetical protein blr7360
bsl7372	2.3			hypothetical protein bsl7372
bll7423	1.7			hypothetical protein bll7423
blr7435			1.8	hypothetical protein blr7435
blr7436	1.6		2.2	hypothetical protein blr7436
blr7478	-1.6	5.5		hypothetical protein blr7478
blr7486	2.2			hypothetical protein blr7486
bll7494		1.6		hypothetical protein bll7494
blr7500		1.5		hypothetical protein blr7500
blr7528	1.7			hypothetical protein blr7528
bll7538	1.5		2.7	hypothetical protein bll7538
bll7551	3.4	2.1	1.9	hypothetical protein bll7551
bll7553		1.7		hypothetical protein bll7553
bll7701	2.1			hypothetical protein bll7701
blr7713	2.4	1.5		hypothetical protein blr7713
bll7745		1.5		hypothetical protein bll7745
bll7750		1.7		hypothetical protein bll7750
blr7780	1.8			hypothetical protein blr7780
bll7787	2.5			hypothetical protein bll7787
blr7878	-2.0	1.6		hypothetical protein blr7878
bsl7915			1.8	hypothetical protein bsl7915
blr7950	5.4			hypothetical protein blr7950
bll7987	1.8			hypothetical protein bll7987
bll7990	3.0			hypothetical protein bll7990
bll7991	3.0			hypothetical protein bll7991
bsl7992	2.2		1.8	hypothetical protein bsl7992
bll7993	2.4	3.3		hypothetical protein bll7993
bsl8016	1.9			hypothetical protein bsl8016
bsl8023		1.6		hypothetical protein bsl8023
bll8033	1.5			hypothetical protein bll8033
bll8093	1.8			hypothetical protein bll8093
blr8112		1.7		hypothetical protein blr8112
bsl8228		1.5		hypothetical protein bsl8228
bsr8262		2.4		hypothetical protein bsr8262
bll8277	1.7			hypothetical protein bll8277
bll8283		1.6		hypothetical protein bll8283
bll8307	1.6			hypothetical protein bll8307
bsr8311		1.6		hypothetical protein bsr8311
blr0002		-1.6		hypothetical protein blr0002
bsl0032	-1.8			hypothetical protein bsl0032
bll0163		-1.7		hypothetical protein bll0163
bsl0170		-1.8		hypothetical protein bsl0170
bsr0189		-1.8		hypothetical protein bsr0189
bsl0231		-1.6		hypothetical protein bsl0231

bll0233		-1.6		hypothetical protein bll0233
bll0235		-1.5		hypothetical protein bll0235
blr0306		-2.1		hypothetical protein blr0306
bll0362	-1.6			hypothetical protein bll0362
bll0403	-1.5			hypothetical protein bll0403
bll0427		-1.9		hypothetical protein bll0427
blr0475	-1.5			hypothetical protein blr0475
bll0507		-3.2	-1.9	hypothetical protein bll0507
bll0544		-1.8		hypothetical protein bll0544
bll0555		-2.3	-1.7	hypothetical protein bll0555
blr0586	-1.9			hypothetical protein blr0586
bll0599		-1.9		hypothetical protein bll0599
blr0628			-1.7	hypothetical protein blr0628
bll0645		-1.7		hypothetical protein bll0645
blr0721		-2.0		hypothetical protein blr0721
blr0787		-1.5		hypothetical protein blr0787
bll0805	-2.3			hypothetical protein bll0805
bll0824	-1.9			hypothetical protein bll0824
blr0845.3n	-1.6			hypothetical protein blr0845.3n
blr0857.2n		-1.9		hypothetical protein blr0857
bll0863		-1.6		hypothetical protein bll0863
bsr0959		-1.8		hypothetical protein bsr0959
blr0960	-1.6			hypothetical protein blr0960
blr0978		-1.7		hypothetical protein blr0978
bll1026		-1.5		hypothetical protein bll1026
bll1126	-1.5			hypothetical protein bll1126
blr1245		-1.7		hypothetical protein blr1245
blr1261		-1.6		hypothetical protein blr1261
blr1283		-1.7		hypothetical protein blr1283
blr1303		-1.7		hypothetical protein blr1303
blr1330		-6.0		hypothetical protein blr1330
blr1332		-1.5		hypothetical protein blr1332
bsl1334.1n		-1.6		hypothetical protein bsl1334
bll1335		-1.5		hypothetical protein bll1335
blr1347		-2.0		hypothetical protein blr1347
bll1362	-1.7	-5.5		hypothetical protein bll1362
bsl1363		-1.7		hypothetical protein bsl1363
bll1401		-2.3		hypothetical protein bll1401
bsl1405			-1.8	hypothetical protein bsl1405
blr1408		-1.5		hypothetical protein blr1408
bll1462		-1.7		hypothetical protein bll1462
bll1465	1.5	-1.5		hypothetical protein bll1465
bll1466		-1.9		hypothetical protein bll1466
bll1467	-1.7			hypothetical protein bll1467
blr1469		-2.0		hypothetical protein blr1469
bsl1473		-1.7		hypothetical protein bsl1473
blr1480		-2.0		hypothetical protein blr1480
bsr1505		-1.9		hypothetical protein bsr1505
bsr1514		-1.5		hypothetical protein bsr1514
bsr1561		-1.5		hypothetical protein bsr1561
bll1587	-5.4	-1.5		hypothetical protein bll1587
bll1594	-1.6			hypothetical protein bll1594
blr1627		-3.9		hypothetical protein blr1627
blr1640	-1.7			hypothetical protein blr1640
blr1704		-1.8		hypothetical protein blr1704
bsr1831		-1.5		hypothetical protein bsr1831

blr1839	-1.5			hypothetical protein blr1839
bll1844	-2.0			hypothetical protein bll1844
blr1867	-1.6			hypothetical protein blr1867
blr1879	-1.7			hypothetical protein blr1879
bsr1909		-1.7		hypothetical protein bsr1909
bll1942		-1.5		hypothetical protein bll1942
bll1944	-2.0			hypothetical protein bll1944
bll1948		-1.8		hypothetical protein bll1948
bll1980	-1.6			hypothetical protein bll1980
bll1981	-1.7	-1.9		hypothetical protein bll1981
bll2009	-1.8	-1.7		hypothetical protein bll2009
bsr2013		-2.7		hypothetical protein bsr2013
bsl2014	-1.7			hypothetical protein bsl2014
bll2087		-1.5		hypothetical protein bll2087
blr2202		-1.8		hypothetical protein blr2202
blr2242	-1.5			hypothetical protein blr2242
blr2259	-1.6			hypothetical protein blr2259
bll2284		-1.6		hypothetical protein bll2284
blr2286		-1.5		hypothetical protein blr2286
bll2323	-1.5			hypothetical protein bll2323
bll2357	-1.5			hypothetical protein bll2357
blr2359	-1.6			hypothetical protein blr2359
bll2361	-1.8			hypothetical protein bll2361
bsr2400		-2.0		hypothetical protein bsr2400
blr2408	-1.6			hypothetical protein blr2408
bll2420	-2.2			hypothetical protein bll2420
bll2452		-1.7		hypothetical protein bll2452
bll2537		-1.8		hypothetical protein bll2537
blr2571		-2.6		hypothetical protein blr2571
bsl2593		-1.8	-1.6	hypothetical protein bsl2593
bsl2596		-2.1		hypothetical protein bsl2596
blr2597		-1.5		hypothetical protein blr2597
bll2600		-1.6		hypothetical protein bll2600
blr2614		-1.5		hypothetical protein blr2614
bll2627		-1.7		hypothetical protein bll2627
bll2650		-2.3		hypothetical protein bll2650
blr2669		-1.7		hypothetical protein blr2669
blr2702	-1.7			hypothetical protein blr2702
blr2728		-2.4		hypothetical protein blr2728
blr2751		-1.6		hypothetical protein blr2751
blr2756		-1.8		hypothetical protein blr2756
blr2823		-2.1		hypothetical protein blr2823
bll2902	-1.5			hypothetical protein bll2902
blr2932		-1.5		hypothetical protein blr2932
blr2944			-2.6	hypothetical protein blr2944
blr2945		-4.8		hypothetical protein blr2945
bll2959	-1.7	-3.0		hypothetical protein bll2959
blr3080		-1.7		hypothetical protein blr3080
bsl3119		-1.6		hypothetical protein bsl3119
blr3331		-1.8		hypothetical protein blr3331
blr3364		-2.8		hypothetical protein blr3364
bll3387		-4.2		hypothetical protein bll3387
blr3398		-1.5		hypothetical protein blr3398
bll3421		-1.6		hypothetical protein bll3421
bll3489		-1.7		hypothetical protein bll3489
blr3496			-1.6	hypothetical protein blr3496

blr3497		-1.5		hypothetical protein blr3497
blr3550	-1.5			hypothetical protein blr3550
blr3572		-2.7	-1.7	hypothetical protein blr3572
bll3610		-1.7		hypothetical protein bll3610
bll3635		-1.8		hypothetical protein bll3635
bsl3676		-1.7		hypothetical protein bsl3676
blr3681	-1.6			hypothetical protein blr3681
bsl3716	-2.6	-2.8		hypothetical protein bsl3716
bsl3786		-1.5		hypothetical protein bsl3786
blr3798		-1.7		hypothetical protein blr3798
bsr3833		-1.9		hypothetical protein bsr3833
bll3836			-1.8	hypothetical protein bll3836
bll3837		-3.0		hypothetical protein bll3837
blr3848		-4.2		hypothetical protein blr3848
blr3860	-1.7	-2.1		hypothetical protein blr3860
blr3862	-1.8			hypothetical protein blr3862
blr3898	-1.7			hypothetical protein blr3898
blr3934		-1.6		hypothetical protein blr3934
blr3941			-1.6	hypothetical protein blr3941
bll3976	-1.8			hypothetical protein bll3976
bsl4014	1.5	-1.6		hypothetical protein bsl4014
blr4026		-1.5		hypothetical protein blr4026
blr4035	-1.5			hypothetical protein blr4035
blr4042			-1.6	hypothetical protein blr4042
blr4063		-1.8		hypothetical protein blr4063
blr4066		-1.5		hypothetical protein blr4066
blr4073	-1.6			hypothetical protein blr4073
blr4098		-1.6		hypothetical protein blr4098
bll4141	-1.5			hypothetical protein bll4141
blr4155	-1.9		-1.5	hypothetical protein blr4155
bll4177	-1.6	-1.5		hypothetical protein bll4177
blr4231			-2.0	hypothetical protein blr4231
bsr4235		-1.7		hypothetical protein bsr4235
blr4279		-4.1	-1.6	hypothetical protein blr4279
blr4310		-1.7		hypothetical protein blr4310
blr4317			-1.7	hypothetical protein blr4317
blr4345	-1.8			hypothetical protein blr4345
bll4347	-2.3			hypothetical protein bll4347
blr4356		-1.5		hypothetical protein blr4356
blr4383		-1.6		hypothetical protein blr4383
blr4392		-1.5		hypothetical protein blr4392
bsr4406		-1.6		hypothetical protein bsr4406
bsl4437		-1.7		hypothetical protein bsl4437
blr4451	-2.8			hypothetical protein blr4451
blr4499	-1.6			hypothetical protein blr4499
bsr4529	1.8	-3.2		hypothetical protein bsr4529
blr4530		-1.5		hypothetical protein blr4530
blr4532		-1.7		hypothetical protein blr4532
bll4537		-2.0		hypothetical protein bll4537
blr4539	-1.9			hypothetical protein blr4539
blr4566	-1.5	-2.7		hypothetical protein blr4566
bll4579		-1.6		hypothetical protein bll4579
bsl4610	-1.5			hypothetical protein bsl4610
blr4629		-2.2		hypothetical protein blr4629
bsr4666	-1.6			hypothetical protein bsr4666
bsr4668	-1.5			hypothetical protein bsr4668

blr4675.2n		-2.1		hypothetical protein blr4675
blr4676		-1.6		hypothetical protein blr4676
bll4713	-1.6			hypothetical protein bll4713
bll4722	-2.5			hypothetical protein bll4722
bll4733		-1.9		hypothetical protein bll4733
blr4737		-2.2		hypothetical protein blr4737
bll4788	-1.8			hypothetical protein bll4788
bll4793		-1.7		hypothetical protein bll4793
blr4840		-2.5		hypothetical protein blr4840
bll4985	-1.7			hypothetical protein bll4985
bll5004	-1.7			hypothetical protein bll5004
bsl5034		-1.7		hypothetical protein bsl5034
bll5040	-2.6	-1.8		hypothetical protein bll5040
bll5085		-1.6		hypothetical protein bll5085
bll5130		-2.4		hypothetical protein bll5130
blr5152	-1.7			hypothetical protein blr5152
blr5213	-1.6			hypothetical protein blr5213
bll5287	-1.6			hypothetical protein bll5287
blr5292	-1.5			hypothetical protein blr5292
blr5325	-1.5	-1.7		hypothetical protein blr5325
blr5341		-1.7		hypothetical protein blr5341
blr5432		-1.7		hypothetical protein blr5432
bll5458		-1.6		hypothetical protein bll5458
bll5483	-1.6			hypothetical protein bll5483
bsr5487			-1.6	hypothetical protein bsr5487
blr5489	-2.0			hypothetical protein blr5489
bsl5490		-1.8		hypothetical protein bsl5490
bll5494		-1.8		hypothetical protein bll5494
bll5501		-2.7		hypothetical protein bll5501
blr5502		-2.5		hypothetical protein blr5502
bll5505	-1.6			hypothetical protein bll5505
bsr5508		-1.7		hypothetical protein bsr5508
blr5512	-1.5			hypothetical protein blr5512
blr5533		-2.5		hypothetical protein blr5533
blr5546	-1.6			hypothetical protein blr5546
blr5550		-1.7		hypothetical protein blr5550
bll5559	-1.9	-1.8		hypothetical protein bll5559
blr5564		-2.0		hypothetical protein blr5564
blr5568		-1.9		hypothetical protein blr5568
blr5583	-2.0	-1.7		hypothetical protein blr5583
bsr5590	-1.6			hypothetical protein bsr5590
blr5614		-2.9		hypothetical protein blr5614
bll5643	-1.6			hypothetical protein bll5643
bll5695		-2.0		hypothetical protein bll5695
bsl5717		-1.7		hypothetical protein bsl5717
bsr5760.4n		-1.8		hypothetical protein bsr5760
bll5764		-1.5		hypothetical protein bll5764
blr5815		-1.7		hypothetical protein blr5815
bll5822		-2.9		hypothetical protein bll5822
bll5844		-1.6		hypothetical protein bll5844
bll5846		-1.7		hypothetical protein bll5846
bll5852		-2.8		hypothetical protein bll5852
blr5858		-1.5		hypothetical protein blr5858
blr5884		-1.5		hypothetical protein blr5884
blr5897			-1.6	hypothetical protein blr5897
bll5926		-2.0		hypothetical protein bll5926

blr5992		-1.6		hypothetical protein blr5992
bsl6114		-2.0		hypothetical protein bsl6114
bsl6119	-1.6			hypothetical protein bsl6119
blr6123		-1.7		hypothetical protein blr6123
bll6124	-1.5			hypothetical protein bll6124
blr6140	-1.6			hypothetical protein blr6140
blr6172	-2.3			hypothetical protein blr6172
bll6178		-2.4		hypothetical protein bll6178
bsr6193		-1.6		hypothetical protein bsr6193
blr6269		-1.8		hypothetical protein blr6269
bsl6289		-1.9		hypothetical protein bsl6289
bll6314		-1.5		hypothetical protein bll6314
bll6436		-1.5		hypothetical protein bll6436
bsr6440		-1.5		hypothetical protein bsr6440
bsr6466		-1.6		hypothetical protein bsr6466
bsl6507		-1.6		hypothetical protein bsl6507
bll6514		-2.1		hypothetical protein bll6514
bsl6560		-2.5		hypothetical protein bsl6560
bll6612		-3.4		hypothetical protein bll6612
bll6615		-1.6		hypothetical protein bll6615
blr6624	-1.5			hypothetical protein blr6624
blr6629		-1.7		hypothetical protein blr6629
bll6640			-2.1	hypothetical protein bll6640
blr6643		-1.7		hypothetical protein blr6643
bll6670		-1.6		hypothetical protein bll6670
bll6688	-1.5			hypothetical protein bll6688
bll6691		-3.2		hypothetical protein bll6691
blr6718	-1.6			hypothetical protein blr6718
blr6766		-1.7		hypothetical protein blr6766
blr6774	-1.9	-1.6		hypothetical protein blr6774
blr6789		-3.6	-2.0	hypothetical protein blr6789
bll6808	-1.6			hypothetical protein bll6808
bll6811	-1.7			hypothetical protein bll6811
blr6907		-1.8		hypothetical protein blr6907
bll6916		-1.6		hypothetical protein bll6916
blr6922	-1.8			hypothetical protein blr6922
bsr6998		-1.9		hypothetical protein bsr6998
bll7046			-1.7	hypothetical protein bll7046
bll7047		-1.8		hypothetical protein bll7047
bsr7048		-1.5		hypothetical protein bsr7048
bll7107	-1.6			hypothetical protein bll7107
blr7118		-1.6		hypothetical protein blr7118
blr7140	-1.5			hypothetical protein blr7140
blr7232	-1.6			hypothetical protein blr7232
bll7304		-1.9		hypothetical protein bll7304
blr7305	-1.7			hypothetical protein blr7305
blr7327	-1.7	-4.3		hypothetical protein blr7327
bsr7328	-1.6			hypothetical protein bsr7328
bll7340	-1.8	-1.5		hypothetical protein bll7340
bll7347		-1.5		hypothetical protein bll7347
blr7373	-1.7			hypothetical protein blr7373
bsr7383		-1.5		hypothetical protein bsr7383
bll7399	-2.5			hypothetical protein bll7399
bll7410	-1.9	-2.4		hypothetical protein bll7410
bll7411		-2.4		hypothetical protein bll7411
bll7437	-1.6			hypothetical protein bll7437

blr7447		-2.7		hypothetical protein blr7447
blr7467	-1.6			hypothetical protein blr7467
blr7483	-1.7			hypothetical protein blr7483
bsr7503	-1.9			hypothetical protein bsr7503
bll7512		-1.7		hypothetical protein bll7512
bll7529		-2.1		hypothetical protein bll7529
blr7534		-1.5		hypothetical protein blr7534
blr7542	-1.7	-1.6		hypothetical protein blr7542
blr7567		-2.5		hypothetical protein blr7567
bll7580		-2.1		hypothetical protein bll7580
bsr7643		-1.5		hypothetical protein bsr7643
bll7644		-2.0	-1.7	hypothetical protein bll7644
bll7648		-1.6		hypothetical protein bll7648
bsr7655		-2.6		hypothetical protein bsr7655
blr7656		-1.6		hypothetical protein blr7656
bll7697.2n	-1.8			hypothetical protein bll7697.2n
bll7711		-1.7		hypothetical protein bll7711
bsr7725		-1.7		hypothetical protein bsr7725
bll7735	-1.6			hypothetical protein bll7735
blr7760		-1.5		hypothetical protein blr7760
bll7772	-1.6			hypothetical protein bll7772
bsl7781		-1.5		hypothetical protein bsl7781
blr7794	-1.7			hypothetical protein blr7794
bsr7796		-1.5	-1.7	hypothetical protein bsr7796
bll7911	-1.9	-2.2		hypothetical protein bll7911
blr7978		-1.6		hypothetical protein blr7978
blr8006	-1.6			hypothetical protein blr8006
bll8019	-3.0			hypothetical protein bll8019
bll8034		-2.3		hypothetical protein bll8034
bsr8052	-1.7			hypothetical protein bsr8052
bsl8059	-1.7			hypothetical protein bsl8059
bll8068		-1.5		hypothetical protein bll8068
bll8082		-2.1		hypothetical protein bll8082
blr8088		-1.5		hypothetical protein blr8088
bsl8092	-1.7			hypothetical protein bsl8092
bll8229	-1.8			hypothetical protein bll8229
bsr8246.2n	-1.6			hypothetical protein bsr8246.2n
bll8251	-1.9			hypothetical protein bll8251
bll8274		-1.6		hypothetical protein bll8274

† Significance based on a ≥1.5 (or ≤-1.5) –fold induction cutoff, a false discovery rate of <5 %, and a *q* value of 0.05.

Table S2. *Bradyrhizobium japonicum* USDA 110 genes significantly regulated in response to elevated atmospheric CO_2 concentration growing in nutrient solution without soybean plants.

Locus	Gene	Fold change† (Elevated CO_2/Ambient)	Description
bll0043	*traG*	3.2	conjugal transfer protein
bll0065		4.9	putative DNA-binding protein
bll0472		1.8	probable intracellular septation protein
bll0633	*gidA*	1.7	glucose inhibited division protein A
bll0793		2.3	hypothetical protein bll0793
bll1001		2.0	probable ABC transporter permease protein
bll1057		2.6	ABC transporter permease protein
bll1186	*atpB'*	2.2	FoF1 ATP synthase B' chain
bll4778		2.3	dihydrolipoamide dehydrogenase
bll6486		2.0	hypothetical protein bll6486
bll6674		2.0	hypothetical protein bll6674
bll7125	*pntAB*	1.7	NAD(P) transhydrogenase subunit alpha part 2
bll7226		2.2	hypothetical protein bll7226
blr1356		2.3	peptide ABC transporter ATP-binding protein
blr1614		1.5	hypothetical protein blr1614
blr2451.1n		1.8	hypothetical protein blr2451.1n
blr2768	*fixH*	2.9	FixH protein
blr3770		2.1	hypothetical protein blr3770
blr3898		3.4	hypothetical protein blr3898
blr5093		4.2	transcriptional regulatory protein LysR family
blr5632		3.0	hypothetical glutathione S-transferase like protein
blr5838	*flgI*	2.7	flagellar P-ring protein precursor
blr5872	*gudD*	3.8	glucarate dehydratase
blr6544		3.0	hypothetical protein blr6544
blr7058		2.5	transcriptional regulatory protein TetR family
blr7732		2.5	hypothetical protein blr7732
blr7845		3.5	long-chain-fatty-acid-CoA-ligase
bsl0708	*rpmI*	2.2	50S ribosomal protein L35
bsl2479		3.0	hypothetical protein bsl2479

† Significance based on a $\geq$ 1.5 (or $\leq$ -1.5) –fold induction cutoff, a false discovery rate of <5 %, and a *q* value of 0.05.

Table S3. Significantly up-regulated *Bradyrhizobium japonicum* FixK_2-associated genes in response to elevated atmospheric CO_2 concentration[†]

Locus	Gene	Fold change (Elevated CO_2/Ambient) [‡] USDA 110	SFJ14-36	SFJ4-24	Description
blr0497		1.8	3.5	-	hypothetical protein blr0497
blr1289		2.4	2.7	-	hypothetical protein blr1289
blr1311		-	1.5	-	outer membrane protein
blr1883	$rpoN_1$	1.7	1.6	-	RNA polymerase sigma-54 subunit
bll2007	$hemN_1$	1.8	1.5	-	coproporphyrinogen III dehydrogenase
bll2109		2.4	-	-	transcriptional regulatory protein Crp family
bsl2328		3.4	-	-	hypothetical protein bsl2328
bll2330		1.8	-	-	hypothetical protein bll2330
bll2471		2.0	1.9	-	hypothetical protein bll2471
blr2659		2.9	-	-	hypothetical protein blr2659
bll2662		2.2	-	-	hypothetical protein bll2662
bsr2670		1.6	-	-	hypothetical protein bsr2670
blr2761		2.1	2.0	-	hypothetical protein blr2761
blr2763	*fixN*	1.9	1.5	1.8	cytochrome-c oxidase
blr2764	*fixO*	3.5	-	1.6	cytochrome-c oxidase
bsr2765	*fixQ*	2.9	1.8	2.8	cbb3 oxidase, subunit IV
blr2766	*fixP*	4.4	-	2.2	cbb3 oxidase, subunit III
blr2767	*fixG*	2.3	1.7	4.1	iron-sulfur cluster-binding protein
blr2768	*fixH*	3.0	-	2.5	FixH protein
blr2769	*fixI*	1.7	1.6	-	E1-E2 type cation ATPase
blr2987		1.7	-	-	hypothetical protein blr2987
bll3998	*hcaB*	1.8	3.4	-	Vanillin:oxygen oxidoreductase
blr4114		1.9	2.1	-	hypothetical protein blr4114
blr4115		1.7	1.5	-	putative symporter
blr4174		1.8	-	-	hypothetical protein blr4174
bsr4175		1.6	-	-	hypothetical protein bsr4175
blr4637		2.2	-	-	probable HspC2 heat shock protein
bsl4650		2.7	-	-	hypothetical protein bsl4650
bll4651		3.6	-	-	hypothetical protein bll4651
blr4652		2.7	-	-	hypothetical protein blr4652
blr4653	*dnaJ*	2.8	*-1.6*	-	molecular chaperone, DnaJ family
blr4654		3.1	2.2	-	hypothetical protein blr4654
bsl5002		2.4	-	-	hypothetical protein bsl5002
bsr5273		2.2	-	2.5	hypothetical protein bsr5273
bll5315		2.7	-	4.1	hypothetical protein bll5315
bll5315.1n		-	1.5	2.2	hypothetical protein bll5315
bll5655		1.6	2.6	-	alcohol dehydrogenase
bll6061	$fixK_1$	1.6	-	2.2	transcriptional regulatory protein Crp family
bll6069		1.8	-	1.8	hypothetical protein bll6069
blr6071		2.4	-	-	hypothetical protein blr6071
bll6073	*phbC*	1.6	-	-	probable poly-beta-hydroxybutyrate polymerase
blr6074		1.6	2.9	-	hypothetical protein blr6074
blr6128	*cycB*	2.4	-	-	cytochrome c552
bsr7036	*napE*	2.6	1.7	-	periplasmic nitrate reductase protein
blr7037	*napD*	2.7	-	-	periplasmic nitrate reductase
blr7039	*napB*	3.2	-	-	periplasmic nitrate reductase small subunit precursor
blr7040	*napC*	3.3	-	-	cytochrome C-type protein
bll7086	*hemN*	2.9	2.0	2.6	anaerbic coproporphyrinogen III oxidase
bsr7087		1.7	-	-	hypothetical protein bsr7087
blr7088		2.2	-	1.8	hypothetical protein blr7088
blr7089	*nirK*	2.6	1.8	1.7	respiratory nitrite reductase

bsl7372	2.3	-	-	hypothetical protein bsl7372
blr7345	2.2	-	-	hypothetical protein blr7345
bll7553	-	1.7	-	hypothetical protein bll7553
bll7787	2.5	-	-	hypothetical protein bll7787
blr7961	2.0	1.8	-	probable HspC2 heat shock protein

†Genes originally identified by Mesa et al. (2008).

‡Significance based on a ≥1.5 (or ≤ -1.5) – fold induction cutoff, a false discovery rate of <5 %, and a *q* value of 0.05.
